# Linkage disequilibrium and haplotype block patterns in popcorn populations

**DOI:** 10.1371/journal.pone.0219417

**Published:** 2019-09-25

**Authors:** Andréa Carla Bastos Andrade, José Marcelo Soriano Viana, Helcio Duarte Pereira, Vitor Batista Pinto, Fabyano Fonseca e Silva

**Affiliations:** 1 Federal University of Viçosa, Department of General Biology, Viçosa, MG, Brazil; 2 Federal University of Viçosa, Department of Animal Science, Viçosa, MG, Brazil; North Dakota State University, UNITED STATES

## Abstract

Linkage disequilibrium (LD) analysis provides information on the evolutionary aspects of populations. Recently, haplotype blocks have been used to increase the power of quantitative trait loci detection in genome-wide association studies and the prediction accuracy of genomic selection. Our objectives were as follows: to compare the degree of LD, LD decay, and LD decay extent in popcorn populations; to characterize the number and length of haplotype blocks in the populations; and to determine whether maize chromosomes also have a pattern of interspaced regions of high and low rates of recombination. We used a biparental population, a synthetic, and a breeding population, genotyped for approximately 75,000 single nucleotide polymorphisms (SNPs). The sample size ranged from 190 to 192 plants. For the whole-genome LD and haplotype block analyses, we assumed a window of 500 kb. To characterize the block and step patterns of LD in the populations, we constructed LD maps by chromosome, defining a cold spot as a chromosome segment including SNPs with the same LDU position. The LD and haplotype block analyses were also performed at the intragenic level, selecting 12 genes related to zein, starch, cellulose, and fatty acid biosynthesis. The populations with the higher and lower frequencies of |D'| values greater than 0.75 were the biparental (65–74%) and the breeding population (26–58%), respectively. There were slight differences between the populations regarding the average distance for SNPs with |D'| values greater than 0.75 (in the range of approximately 207 to 229 kb). The level of LD expressed by the r^2^ values was low in the populations (0.02, 0.04, and 0.04, on average) but comparable to some non-isolated human populations. The frequency of r^2^ values greater than 0.75 was lower in the biparental population (0.2–0.5%) and higher in the other populations (0.2–1.6%). The average distance for SNPs with r^2^ values greater than 0.75 was much higher in the biparental population (approximately 80 to 126 kb). In the other populations, the ranges were approximately 6 to 19 and 6 to 35 kb. The heatmaps for the regions covered by the first 100 SNPs in each chromosome, in each population (1 to 3.3 Mb, approximately), provided evidence that the comparatively few high r^2^ values (close to 1.0) occurred only for SNPs in close proximity, especially in the synthetic and breeding populations. Due to the reduced number of SNPs in the haplotype blocks (2 to 3) in the populations, it is not expected advantage of a haplotype-based association study as well as genomic selection along generations. The results concerning LD decay (rapid decay after 5–10 kb) and LD decay extent (along up to 300 kb) are in the range observed with maize inbred line panels. The LD maps indicate that maize chromosomes had a pattern of regions of extensive LD interspaced with regions of low LD. However, our simulated LD map provides evidence that this pattern can reflect regions with differences in allele frequencies and LD levels (expressed by |D'|) and not regions with high and low rates of recombination.

## Introduction

Linkage disequilibrium (LD) analysis is important to humans, other animal species, and plant geneticists because the results can be used for positional cloning, provide information on the rate of recombination, gene conversion, and evolutionary aspects of populations, including recombination history, mutation, selection, genetic drift, and admixture, and allow for the selection of populations and single nucleotide polymorphisms (SNPs) for association studies [1]. The most common LD measures are D' and r^2^. The statistic D' is the ratio between D (the difference between products of haplotypes, D = P(AB).P(ab)–P(Ab).P(aB)) and the deviation of the actual gametic frequency from linkage equilibrium [2]. The statistic r^2^ is the square of the correlation between the values of alleles at two loci in the same gamete, where D is the covariance [3].

Additional information on historical recombination is provided by analysis of the haplotype block pattern in populations. A haplotype block is a chromosome region in which there are few haplotypes (combinations of alleles of multiple SNPs within a haplotype block) (2–4 per block), and for which the LD analysis provides evidence of a low rate of recombination [1]. Recently, haplotype blocks have been used to increase the power of QTL (quantitative trait loci) detection in genome-wide association studies (GWAS) and the prediction accuracy with genomic selection. Based on a panel including 183 maize inbred lines genotyped for 38,000 SNPs, Maldonado *et al*. [4] confirmed the advantage of haplotype-based GWAS for ear and plant height, the ear height/plant height ratio, and leaf angle relative to single SNP analysis. Hess *et al*. [5] observed an increase of up to 5.5% in the accuracy of genomic prediction in an admixed dairy cattle population using fixed-length haplotypes relative to the single SNP approach. Although there are several methods for defining a haplotype block, the most common procedure was proposed by Gabriel *et al*. [6]. Their criterion is that the one-sided upper 95% confidence bound on D' is > 0.98 and the lower bound is > 0.70.

Characterization of the LD and haplotype block patterns in human, domesticated animal, and plant populations has provided variable results concerning the degree of LD, LD decay, LD decay extent, and number and length of the haplotype blocks. Most maize LD studies have been done with inbred line panels. Thirunavukkarasu *et al*. [7] and Truntzler *et al*. [8] observed an overall average r^2^ between 0.23 and 0.61, LD decay after 5–10 kb, and LD extent along 200–300 kb. Faster LD decay and shorter LD extent (less than 4 kb) were observed by Maldonado *et al*. [4]. Higher LD and slower LD decay were observed in biparental and multiparental maize populations [9]. The number and length of haplotype blocks is also highly variable [4, 7].

In several investigations in human populations, the structure of LD was described based on LD maps. In an LD map, each SNP has an LD position in LD units (LDUs). One LDU is the distance in kilobases at which disequilibrium (expressed as the Malecot's prediction of association– ρ) declines to approximately 0.37 of its starting value. Assuming unrelated individuals, ρ equates to the absolute value of D'. The difference between the LD positions of two SNPs divided by the distance in kilobases (d) is the exponential decline of disequilibrium (ε). LDUs share an inverse relationship with the recombination rate. Thus, regions with extensive LD have few LDUs (plateaus or blocks), and regions with many LDUs have high levels of recombination rate (steps). Holes in the LD maps are regions where greater marker density is required to provide a full characterization of the block and step patterns of the LD. Holes are identified by an LD map interval of 3, which is an arbitrary value because disequilibrium is indeterminate for εd > 3 and of doubtful reliability for εd > 2 [10, 11].

Because there is no information on LD and the structure of haplotype blocks in popcorn populations and no LD maps for maize, the objectives of this study were: (1) to compare the degree of LD, the LD decay, and the LD decay extent in popcorn populations; (2) to characterize the number and length of haplotype blocks in the populations; and (3) to elaborate the first LD map for maize, for elucidating whether maize chromosomes also have a pattern of interspaced regions of high and low rates of recombination.

## Materials and methods

### Populations

We used a biparental (F_2_ generation) temperate population, a tropical synthetic (Synthetic UFV), and a tropical breeding population (Beija-Flor cycle 4). A biparental population is the most used maize population for deriving doubled haploids and inbred lines in hybrid breeding. Maize synthetic varieties are used as germplasm sources in breeding programs or as improved populations in developing countries. Theoretically, a biparental population shows LD only for linked genes and molecular markers. In a synthetic there is LD for genes and molecular markers with independent assortment. Because selection can change the LD degree, we also included a breeding population. The biparental population was derived from the single cross AP4502, developed by the Agricultural Alumni Seed Improvement Association, Romney, IN, USA. Synthetic UFV and Beija-Flor cycle 4 (BFc4) were developed by the Federal University of Viçosa (UFV), Minas Gerais, Brazil. The synthetic was derived by random crossings involving 20 elite inbred lines from the tropical population Viçosa and 20 elite inbred lines from the tropical population Beija-Flor. The inbred lines were selected based on expansion volume (a measure of popcorn quality). Beija-Flor cycle 4 was developed after four cycles of half-sib selection based on expansion volume.

### DNA extraction, genotyping-by-sequencing (GBS), SNP calling, data quality control, and imputation

Leaf samples of young plants were collected for DNA extraction. The DNA extraction was performed using the CTAB (cetyl trimethylammonium bromide) protocol with modifications. After quantification, the DNA samples of 574 plants (190 or 192 from each population) were sent to the Institute of Biotechnology at Cornell University (two plates of 95 samples from the biparental population) and Institut de Recherche en Immunologie et en Cancérologie/IRIC at University of Montreal (four plates of 96 samples from the tropical populations) for GBS services based on HiSeq 2500 (paired-end reads of 125 bp) and NextSeq500 (single-end reads of 85 bp), respectively. The SNP variant call services were provided by the Institute of Biotechnology and Omega Bioservices, Norcross, GA, respectively, using B73 version 4 (current version) as the reference genome [12]. After reading the data using the R package vcfR [13], we filtered by missing allele and chromosome. Then, we computed the SNP and genotype call rates and the minor allele frequency (MAF), employing the R package HapEstXXR [14]. After filtering by MAF > 0.01, we imputed based on Beagle [15] using the R package synbreed [16]. The number of SNPs after data quality control and imputation were 145,420, 74,773, and 76,055 for the biparental population, Synthetic UFV, and Beija-Flor c4, respectively. To maintain a similar number of SNPs for the populations, we finally performed a random sampling of 75,000 SNPs from the biparental population.

### LD and haplotype block analyses

For Hardy-Weinberg equilibrium analysis by population and chromosome, the Bonferroni criterion was adopted to keep a global level of significance of 1%. To characterize the block and step patterns of LD in the populations, we constructed LD maps by chromosome using the interval method [17]. We defined a cold spot region as a chromosome segment including SNPs with the same LDU position. To evaluate if the LD maps allow inference of the overall degree of LD by chromosome in the populations, we also processed a simulated data set, generated with *REALbreeding* software (available by request). This software has been recently used in studies on population structure [18], QTL mapping [19], genomic selection [20], and genome-wide association studies [21]. We simulated the genotyping of 200 individuals in a population (generation 0) and 200 individuals in the same population after 10 generations of random crossings (generation 10), for 287 SNPs spanning 298 cM (density of 1 cM) of a single chromosome.

We then evaluated the degree of LD by chromosome in the populations concerning SNPs separated by up to 500 kb, using a two marker expectation-maximization (EM) algorithm [22]. For the whole-genome LD decay and LD decay extent analyses, we computed the average |D'| and r^2^ values, defining intervals of 50 kb (0–50 to 451–500). To define a haplotype block, we adopted the criterion proposed by Gabriel *et al*. [6]. The haplotypes were estimated using an accelerated EM algorithm with a partition-ligation approach [23] to generate phased haplotypes for population frequency [24].

The LD and haplotype block analyses were also performed at the intragenic level. We choose 12 genes related to zein (one), starch (four), cellulose (five), and fatty acid biosynthesis (two) (S1 Table). With two exceptions, the selected genes had at least five SNPs in each population (maximum of 21). For the intragenic LD decay and LD decay extent analyses, we computed the average |D'| and r^2^ values defining intervals of 1 kb (0–1 to 10.1–11 kb). All analyses were performed using LDMAP [17] and Haploview [22]. Heatmaps were generated using the R package pheatmap. To assess the haplotype blocks information, the haplotype files for each population and chromosome were read by a program (*Haplotype blocks summary*) developed in REALbasic 2009 by Prof. José Marcelo Soriano Viana.

## Results

With the exception of chromosome 10 in the breeding population, the number of SNPs was generally in proportion to the chromosome length, providing an SNP density in the range of 23.5 to 44.3 kb (one SNP per 30.0 kb on average) (Table 1). The average MAF was approximately 0.1 regardless of chromosome and population, but the populations differed in their MAF distribution. The biparental population had a bimodal distribution and showed a higher number of SNPs with frequencies close to 0.01 and greater than 0.45 (S1 Fig). The synthetic and breeding populations had similar MAF distributions. The analysis of Hardy-Weinberg equilibrium provided evidence that most of the SNPs in the biparental population had a nonsignificant deviation, whereas most of the SNPs in the other populations showed a significant deviation. We retained SNPs with significant deviation from Hardy-Weinberg equilibrium in the synthetic and breeding populations to keep a similar number of SNPs for the LD and haplotype block analyses. To maintain a similar number of SNPs for constructing the LD maps by chromosome, we used the SNPs in Hardy-Weinberg equilibrium in the synthetic and breeding populations as well as a sample of SNPs with no significant deviation from Hardy-Weinberg equilibrium from the biparental population.

**Table 1:** Number of SNPs, SNP coverage (kb), average SNP interval (bp) and MAF, and minimum, average, and maximum LD measures by chromosome in each population.

Population	Chr.	SNPs	SNP coverage	SNP interval	MAF		|D'|			r^2^	
						Min.	Av.	Max.	Min.	Av.	Max.
Biparental	1	11,816	307,039.27	25,982.75	0.09	0.00	0.78	1.0	0.00	0.023	1.0
	2	8,710	244,412.25	28,059.68	0.11	0.00	0.77	1.0	0.00	0.026	1.0
	3	8,205	235,520.19	28,693.18	0.11	0.00	0.75	1.0	0.00	0.032	1.0
	4	8,081	246,827.22	30,525.85	0.07	0.00	0.81	1.0	0.00	0.015	1.0
	5	8,697	223,657.67	25,708.94	0.09	0.00	0.79	1.0	0.00	0.019	1.0
	6	5,883	173,906.61	29,537.18	0.10	0.00	0.78	1.0	0.00	0.027	1.0
	7	6,401	182,200.48	28,440.64	0.11	0.00	0.77	1.0	0.00	0.025	1.0
	8	6,528	181,042.64	27,725.54	0.10	0.00	0.78	1.0	0.00	0.023	1.0
	9	5,625	159,429.26	28,336.11	0.11	0.00	0.76	1.0	0.00	0.027	1.0
	10	5,054	150,832.73	29,824.39	0.10	0.00	0.76	1.0	0.00	0.025	1.0
Synthetic	1	11,224	306,909.66	27,341.76	0.10	0.00	0.75	1.0	0.02	0.046	1.0
	2	9,712	244,369.34	25,159.97	0.10	0.00	0.75	1.0	0.02	0.041	1.0
	3	9,374	235,478.72	25,083.00	0.10	0.00	0.76	1.0	0.02	0.042	1.0
	4	5,840	246,943.47	42,170.02	0.10	0.00	0.74	1.0	0.02	0.052	1.0
	5	9,460	223,706.51	23,589.54	0.10	0.00	0.74	1.0	0.02	0.040	1.0
	6	5,294	173,221.42	32,692.62	0.10	0.00	0.74	1.0	0.02	0.050	1.0
	7	6,299	182,159.80	28,857.92	0.11	0.00	0.74	1.0	0.02	0.042	1.0
	8	6,248	180,660.38	28,850.52	0.10	0.00	0.76	1.0	0.02	0.044	1.0
	9	5,161	159,553.33	30,909.31	0.11	0.00	0.75	1.0	0.02	0.045	1.0
	10	6,161	150,828.61	24,464.31	0.09	0.00	0.75	1.0	0.02	0.034	1.0
BFc4	1	10,182	306,774.01	30,126.80	0.11	0.20	0.71	1.0	0.02	0.047	1.0
	2	8,481	244,407.97	28,816.88	0.11	0.21	0.69	1.0	0.02	0.042	1.0
	3	8,005	235,478.74	29,373.18	0.11	0.20	0.70	1.0	0.02	0.040	1.0
	4	5,558	246,840.44	44,379.59	0.11	0.20	0.69	1.0	0.02	0.054	1.0
	5	7,674	223,706.51	29,080.32	0.11	0.20	0.70	1.0	0.02	0.039	1.0
	6	4,547	173,351.50	38,093.29	0.11	0.19	0.68	1.0	0.02	0.044	1.0
	7	5,602	182,155.19	32,448.24	0.11	0.20	0.69	1.0	0.02	0.040	1.0
	8	5,020	180,660.38	35,943.93	0.12	0.20	0.70	1.0	0.02	0.048	1.0
	9	5,353	159,489.87	29,788.60	0.11	0.20	0.69	1.0	0.02	0.042	1.0
	10	15,633	150,926.35	9,653.39	0.13	0.20	0.52	1.0	0.02	0.021	1.0

**Fig 1:**
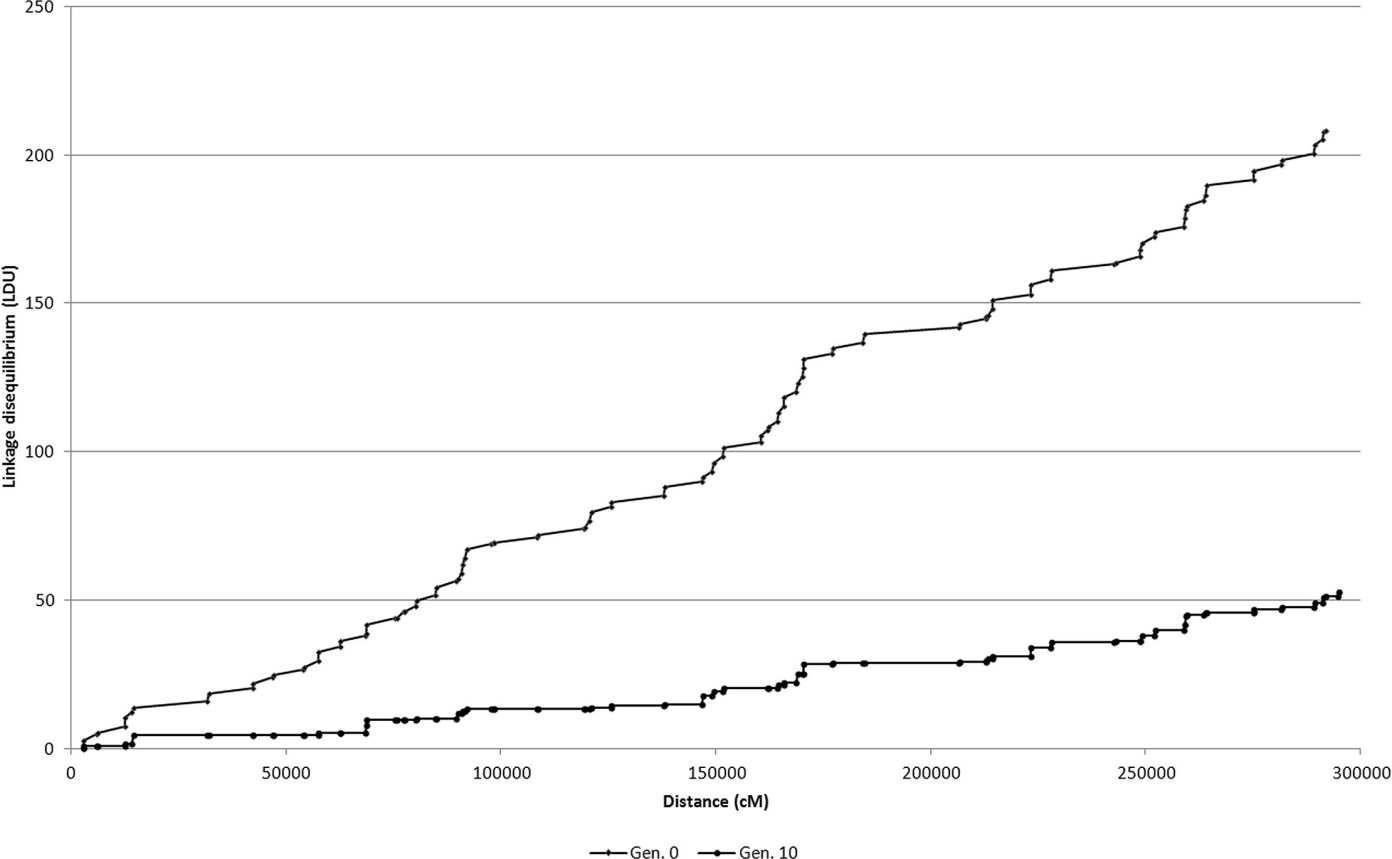
LD maps for generations 0 and 10.

The LD map from the simulated data provided evidence that the LD units were lower for the generation with lower LD (generation 10) (Fig 1). Thus, the LD maps by chromosome revealed that the higher global LD (in LDUs) was observed in the synthetic but only for chromosomes 1 to 7 (S2 Fig). The higher global LD for chromosomes 8 and 9 was observed in the biparental population. The higher global LD for chromosome 10 was seen in the breeding population. The lowest global LD was observed in chromosome 6, and the highest global LD was observed in chromosome 10 of the breeding population. Because of the much higher number of SNPs in Hardy-Weinberg equilibrium in the biparental population, we only used this population for analysis of the number and length of the hot (high recombination rate) and cold (low recombination rate) spot regions of the chromosomes, as well as the number and length of the holes (Table 2). Except for chromosome 10, where the average lengths of the hot and cold spot regions were approximately 37 and 38 kb, respectively, the average lengths of the hot and cold spots regions for the other chromosomes ranged between approximately 45–55 and 83–110 kb, respectively. The number of hot spots ranged between 1,788 and 3,897, and the number of cold spots ranged from 608 to 1,507. The holes represented only 0.4 to 2.7% of the chromosomal genomes.

**Table 2:** Number and minimum, average, and maximum length (kb) of the hot spots (steps), holes, and cold spots (plateaus) by chromosome in the biparental population.

Chr.	Hot	Holes	Cold	Hot spot length			Hole length			Cold spot length		
	spots		spots	Min.	Av.	Max.	Min.	Av.	Max.	Min.	Av.	Max.
1	3897	6	1507	0.001	45.839	1759.096	54.917	194.277	326.226	0.001	84.309	1632.212
2	2691	15	1008	0.001	51.727	1616.834	0.200	204.446	427.934	0.001	101.295	1745.439
3	2541	7	1024	0.001	52.602	2163.519	0.120	185.774	499.444	0.001	98.081	2130.732
4	2868	13	1096	0.001	52.626	1873.436	0.860	241.479	480.843	0.001	84.467	2388.138
5	2822	13	1132	0.001	45.136	2642.230	33.762	189.326	421.771	0.001	82.798	2015.799
6	1892	10	766	0.001	54.869	2872.957	0.053	217.741	433.719	0.001	88.443	1845.273
7	1908	25	749	0.001	50.956	1983.409	0.100	193.875	492.714	0.001	106.740	1014.346
8	1987	14	785	0.001	46.554	1040.453	0.097	162.792	492.395	0.001	109.786	1516.706
9	1788	4	687	0.001	50.341	1362.155	86.562	305.480	498.082	0.001	99.168	1664.406
10	3360	18	608	0.001	37.165	3159.567	3.594	152.031	483.615	0.001	38.306	360.908

Concerning SNPs separated by up to 500 kb, the biparental population and the synthetic had similar average |D'| values (0.77 and 0.75). The values were approximately 10–14% greater than the average value in the breeding population (Table 1). Interestingly, the average r^2^ value in the biparental population was approximately half of the corresponding average values observed in the other populations (0.02 versus 0.04, and 0.04). Regardless of the chromosome, the populations with the higher and lower frequencies of |D'| values greater than 0.75 were the biparental population (65–74%) and the breeding population (26–58%), respectively. However, the frequency of r^2^ values greater than 0.75 was lower in the biparental population (0.2–0.5%) and higher in the other populations (0.2–1.6%) (S2 Table). Furthermore, the average distance for SNPs with r^2^ values greater than 0.75 was much higher in the biparental population (approximately 80 to 126 kb). In the other populations, the ranges were approximately 6 to 19 and 6 to 35 kb. There were slight differences between the populations regarding the average distance for SNPs with |D'| values greater than 0.75 (in the range of approximately 207 to 229 kb).

The heatmaps for the regions covered by the first 100 SNPs in each chromosome, in each population (1 to 3.3 Mb, approximately), provided evidence that the comparatively few high r^2^ values (close to 1.0) occurred only for SNPs in close proximity, especially in the synthetic and breeding populations (S3 Fig). Although these regions do not represent the pattern of LD along the chromosomes (see the LD pattern for five segments of 100 SNPs along chromosome 4 in the biparental population in S4 Fig) there are some regions with blocks of intermediate r^2^ values for distant SNPs, especially in the biparental population.

**Fig 2:**
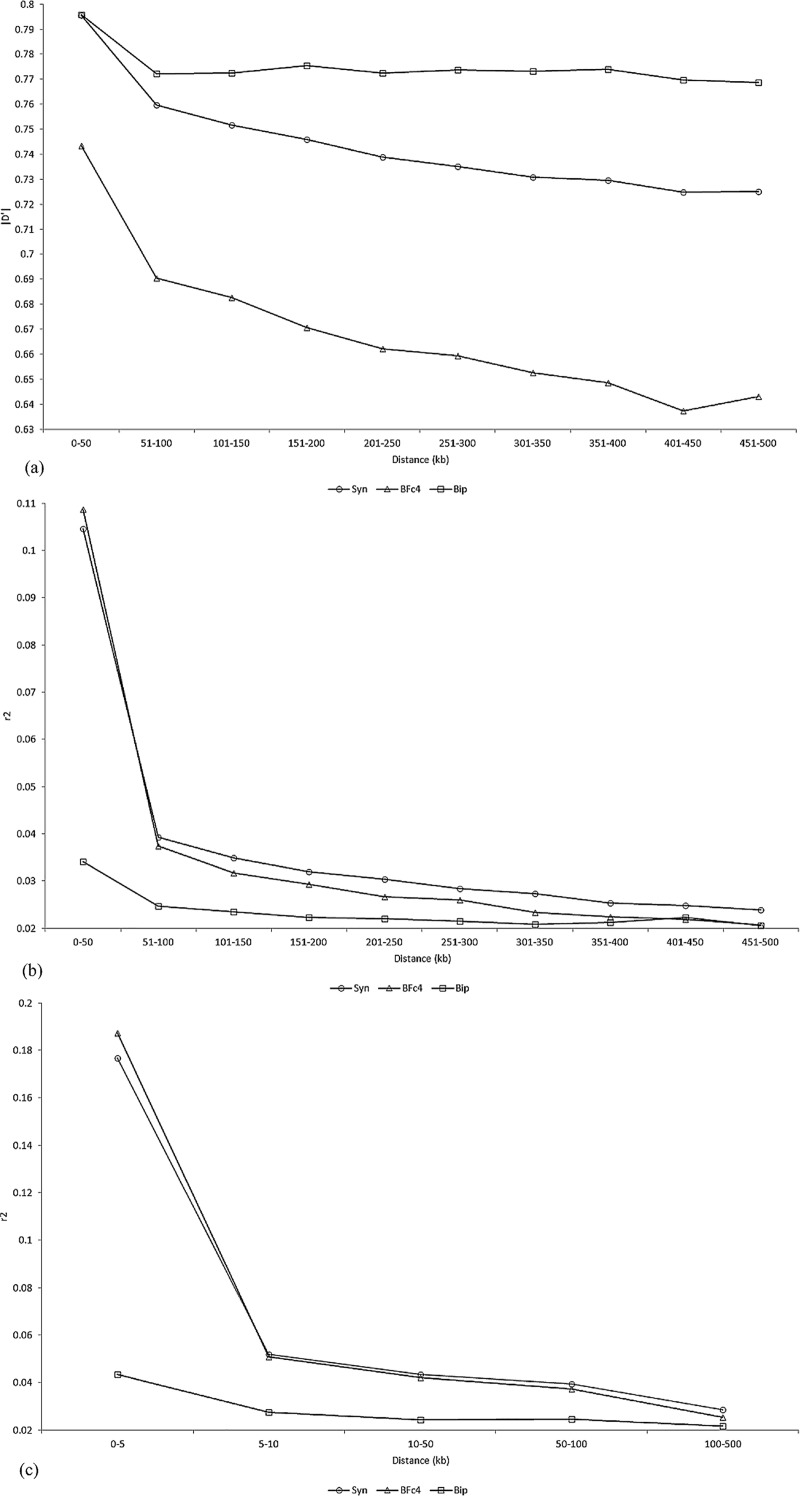
Overall average |D'| (a) and r^2^ (b and c) values by distance interval (kb) in the biparental population (Bip), in the synthetic (Syn), and in the breeding population (BFc4).

Regardless of the chromosome, population, and LD measurement, the LD decreased as the between-SNP distance increased from 0–50 to 451–500 kb (S5 and S6 Figs). In general, there was an initially higher LD decrease for SNPs separated by 51–100 kb (3 to 7% for |D'| and 28 to 66% for r^2^, on average) and then a gradual decrease to the minimum LD value for SNPs separated by 451–500 kb. Because there were no significant differences between chromosomes, we can state that following an initial higher decrease after 50 kb, the |D'| and r^2^ in the biparental population extended with similar magnitude for an interval of 450 kb (Fig 2A and 2B). In this interval, the average |D'| values decreased from 0.69–0.77 to 0.64–0.77 in the three populations, and the average r^2^ values in the biparental population decreased from 0.025 to 0.020. However, in the other two populations, the average r^2^ value decreased by approximately 50%. The r^2^ decay from its maximum average value reached 36 to 73% after 5–10 kb (Fig 2C).

The biparental population also differed from the other populations concerning the pattern of haplotype blocks (Table 3). The biparental population presented a lower average number of haplotype blocks per chromosome (approximately 225 versus 700 and 730 on average), a lower block length (approximately 1 versus 11 kb on average), and a lower number of SNPs per block (approximately 2 versus 3 on average). Most of the haplotype blocks in the three populations included two SNPs, but the number of haplotype blocks with three or more SNPs was greater in the synthetic and breeding populations (S7 Fig). It is important to highlight that the total length of the haplotype blocks represents only 0.01 to 5.13% of the chromosome genomes.

**Table 3:** Haplotype blocks structure of the populations.

Population	Chr.	Blocks	Block size (kb)				SNPs			
			Total	Mean	Min.	Max.	Total	Mean	Min.	Max.
Biparental	1	336	58.60	0.17	0.001	10.30	727	2.2	2	5
	2	294	588.31	2.00	0.001	298.90	647	2.2	2	6
	3	273	307.66	1.13	0.001	101.90	622	2.3	2	5
	4	193	35.80	0.19	0.001	23.15	430	2.2	2	6
	5	218	47.49	0.22	0.001	20.39	484	2.2	2	4
	6	169	419.24	2.48	0.001	292.35	387	2.3	2	5
	7	215	45.60	0.21	0.001	11.68	479	2.2	2	5
	8	186	511.79	2.75	0.001	423.79	409	2.2	2	5
	9	195	58.19	0.29	0.001	15.58	432	2.2	2	5
	10	170	314.88	1.85	0.001	307.49	370	2.2	2	4
Synthetic	1	1126	11935.23	10.60	0.001	494.94	3093	2.7	2	10
	2	935	8501.15	9.09	0.001	451.74	2565	2.7	2	11
	3	810	9065.75	11.19	0.001	457.30	2257	2.8	2	11
	4	525	6615.63	12.60	0.001	423.71	1409	2.7	2	12
	5	933	6428.48	6.89	0.001	395.79	2527	2.7	2	11
	6	496	5051.01	10.18	0.001	492.95	1354	2.7	2	11
	7	569	5169.26	9.09	0.001	317.07	1594	2.8	2	15
	8	583	8927.76	15.31	0.001	476.37	1574	2.7	2	10
	9	486	6553.37	13.48	0.001	398.72	1375	2.8	2	9
	10	534	3905.24	7.31	0.001	434.32	1477	2.8	2	10
BFc4	1	1019	14352.62	14.09	0.001	499.04	2818	2.8	2	12
	2	861	7904.79	9.18	0.001	415.28	2432	2.8	2	11
	3	796	8682.69	10.91	0.001	418.18	2153	2.7	2	16
	4	539	6605.65	12.26	0.001	442.01	1492	2.8	2	12
	5	776	10870.44	14.01	0.001	479.50	2201	2.8	2	15
	6	476	5833.85	12.26	0.001	466.82	1278	2.7	2	7
	7	570	4471.35	7.84	0.001	479.70	1612	2.8	2	13
	8	491	9272.30	18.89	0.001	495.26	1390	2.8	2	12
	9	541	5188.65	9.59	0.001	449.77	1478	2.7	2	8
	10	1236	6619.87	5.36	0.001	471.30	3371	2.7	2	12

**Fig 3:**
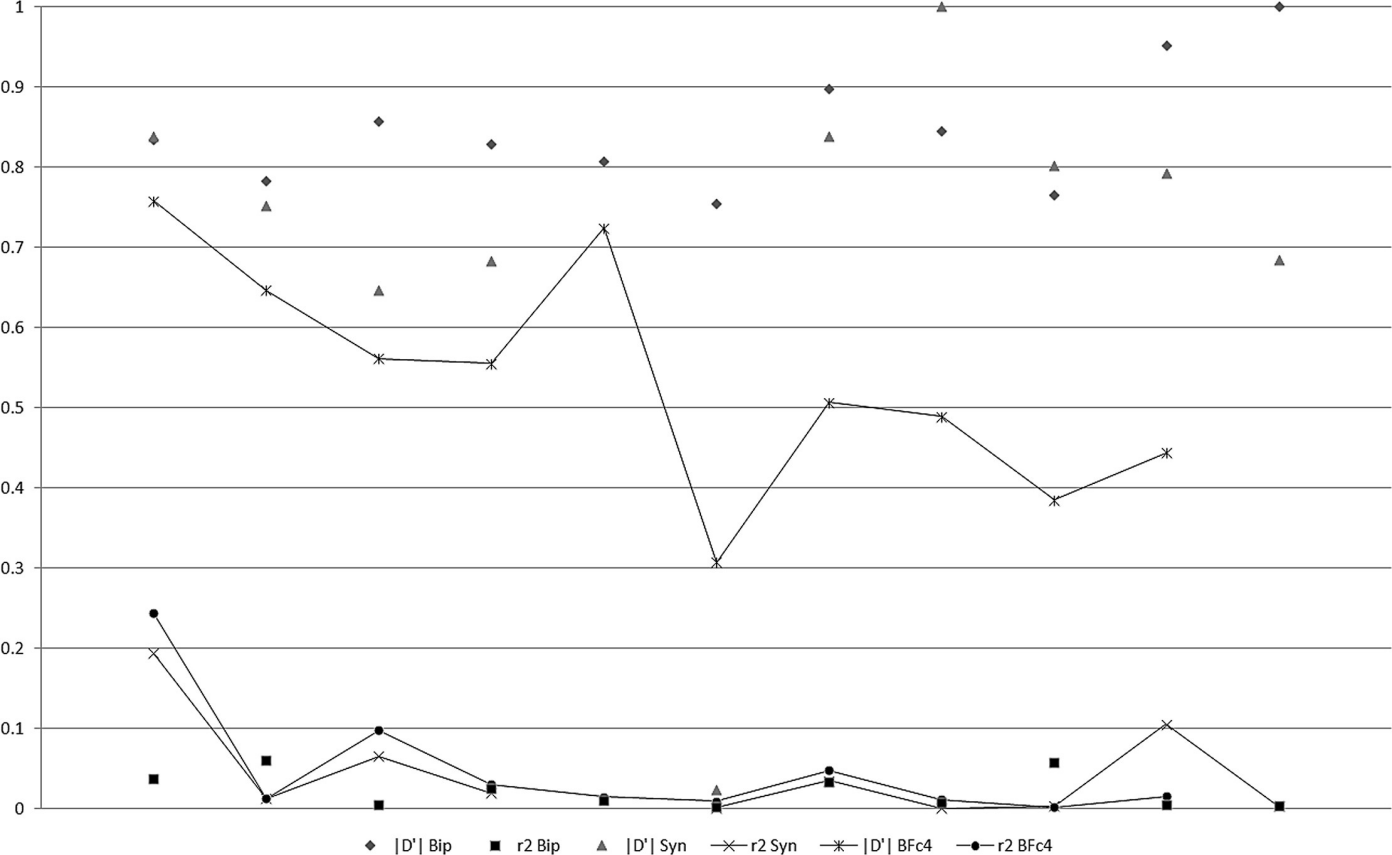
Intragenic LD decay and LD extent concerning SNPs separated by up to 10.6 kb (|D'| and r^2^ average values in intervals of 1 kb).

The intragenic LD analysis also revealed higher average |D'| values in the biparental population and synthetic relative to the average value observed in the breeding population (0.74 and 0.88 versus 0.67). The biparental population presented an average r^2^ value that was much lower than the average values observed in the other two populations (0.02 versus 0.13 and 0.14) (Table 4). Regardless of the population, the maximum intragenic |D'| (1,0) was observed for SNPs separated by up to 10.6 kb, while most of the higher intragenic r^2^ values (0.7 or greater) were only observed for the closest SNPs (S8 and S9 Figs). The intragenic heatmaps provided evidence of distinct LD patterns between genes and populations (S9 Fig). With regard to the intragenic LD decay, there was evidence of |D'| and r^2^ decay in the breeding population and r^2^ decay in the synthetic (Fig 3). Concerning the intragenic haplotype block structure, there was general evidence of a single block of variable size (0.03 to 8.72 kb) with two SNPs (Table 5). Genes Zm00001d018033 and Zm00001d041972 showed population differences in terms of block size and number of SNPs.

**Table 4:** Intragenic minimum, average, and maximum LD values in each population.

Gene	Population		|D'|			r^2^	
		Min.	Av.	Max.	Min.	Av.	Max.
Zm00001d002654	Biparental	0.176	0.96	1.0	0.000	0.005	0.19
	Synthetic	0.003	0.60	1.0	0.000	0.159	1.00
	BFc4	0.042	0.44	1.0	0.000	0.258	1.00
Zm00001d004817	Biparental	0.028	0.81	1.0	0.000	0.004	0.06
	Synthetic	0.059	0.62	1.0	0.000	0.089	1.00
	BFc4	1.000	1.00	1.0	0.002	0.310	0.93
Zm00001d005451	Biparental	0.148	0.91	1.0	0.000	0.003	0.01
	Synthetic	0.407	0.89	1.0	0.000	0.106	1.00
	BFc4	0.057	0.51	1.0	0.000	0.211	0.97
Zm00001d041972	Biparental	0.132	0.89	1.0	0.000	0.004	0.06
	Synthetic	0.263	0.79	1.0	0.000	0.191	1.00
	BFc4	0.193	0.88	1.0	0.000	0.280	1.00
Zm00001d052263	Biparental	0.236	0.85	1.0	0.000	0.011	0.06
	Synthetic	0.217	0.93	1.0	0.000	0.116	1.00
	BFc4	0.323	0.87	1.0	0.000	0.085	1.00
Zm00001d018033	Biparental	0.000	0.83	1.0	0.000	0.031	0.87
	Synthetic	0.488	0.97	1.0	0.000	0.025	0.21
	BFc4	0.137	0.77	1.0	0.001	0.070	0.46
Zm00001d035760	Biparental	0.187	0.84	1.0	0.000	0.007	0.06
	Synthetic	1.000	1.00	1.0	0.007	0.007	0.01
	BFc4	0.721	0.72	0.7	0.027	0.027	0.03
Zm00001d036900	Biparental	0.000	0.76	1.0	0.000	0.093	0.88
	Synthetic	0.005	0.77	1.0	0.000	0.026	1.00
	BFc4	0.031	0.60	1.0	0.000	0.019	0.24
Zm00001d021731	Biparental	0.094	0.59	1.0	0.000	0.037	0.68
	Synthetic	0.019	0.58	1.0	0.000	0.282	1.00
	BFc4	0.193	0.57	1.0	0.001	0.248	1.00
Zm00001d023810	Biparental	1.000	1.00	1.0	0.000	0.000	0.00
	Synthetic	0.026	0.76	1.0	0.000	0.093	1.00
	BFc4	0.004	0.48	1.0	0.000	0.066	0.97
Zm00001d025201	Biparental	0.097	0.84	1.0	0.000	0.004	0.06
	Synthetic	0.059	0.59	1.0	0.000	0.368	0.87
	BFc4	0.006	0.68	1.0	0.000	0.061	1.00
Zm00001d026113	Biparental	0.002	0.82	1.0	0.000	0.026	1.00
	Synthetic	0.105	0.81	1.0	0.000	0.057	0.90
	BFc4	0.015	0.52	1.0	0.000	0.073	1.00

**Table 5:** Intragenic haplotype blocks structure in each population.

Population	Gene	Chr.	Blocks	Block size (kb)				SNPs			
				Total	Mean	Min.	Max.	Total	Mean	Min.	Max.
Biparental	Zm00001d018033	5	1	8.72	8.72	8.72	8.72	2	2	2	2
	Zm00001d026113	10	1	0.03	0.03	0.03	0.03	2	2	2	2
Synthetic	Zm00001d002654	2	1	0.05	0.05	0.05	0.05	3	3	3	3
	Zm00001d004817	2	2	0.22	0.11	0.02	0.21	4	2	2	2
	Zm00001d005451	2	1	0.03	0.03	0.03	0.03	2	2	2	2
	Zm00001d036900	3	1	0.06	0.06	0.06	0.06	2	2	2	2
	Zm00001d041972	3	1	0.02	0.02	0.02	0.02	2	2	2	2
BFc4	Zm00001d041972	3	1	2.22	2.22	2.22	2.22	6	6	6	6
	Zm00001d018033	5	1	0.26	0.26	0.26	0.26	2	2	2	2

## Discussion

It is difficult to characterize the LD and haplotype block patterns in two or more unrelated random cross populations based on an LD map and two measures of linkage disequilibrium. Based on studies of the LD pattern in human populations, LD maps demonstrated that the human chromosomes have a pattern of regions of extensive LD (plateaus or cold spots), interspaced with regions of high recombination rate (steps or hot spots) [25, 26]. Both regions are variable in number and length, and cold spots show equal (as assumed in this study) or similar LD in LDUs. The hot spots present distinct LDUs. The same pattern was seen in the LD maps of the chromosomes of the biparental population, elaborated under high density as recommended by Pengelly *et al*. [25]. To better understand the level of LD in the hot and cold spots, we analyzed two extreme segments of the chromosome 1 LD map, including 30 SNPs. Both segments have similar lengths in LDUs (4.1 and 3.6) and kb (970 and 828). The average |D'| was much greater for the SNPs in the seven cold spots (including three to 12 SNPs) relative to the average value for the SNPs in the 21 hot spots (including two to three SNPs) (0.89 versus 0.29). However, this was not verified via the r^2^ statistic (0.004 versus 0.038).

When comparing populations that share a common origin, have a similar effective population size, and did not face an extreme reduction in size (population bottleneck), the statistics D, D', and r^2^ should provide a comparable characterization of the LD pattern if there are similar allele frequencies. If the populations have distinct distributions of allelic frequencies, D' can be used for analyzing the recombination history, and r^2^ should be the choice if recombination and mutation are important factors affecting the LD [1]. However, in the last two decades, most studies on LD in human populations have aimed to select populations and SNPs (tagging SNPs) for association studies [26, 27]. In general, both |D'| and r^2^ have been used [27, 28], and because of their high level of LD, isolated populations have been recommended for association studies [29]. The statistic r^2^ is the most relevant for association mapping because it has a simple inverse relationship with the sample size required to detect association [1]. The use of LD maps and two measures of LD for comparing the popcorn populations provided some contrasting results, but the general evidence is that the synthetic is the population with the higher LD. As expected, the lower average |D'| value in the breeding population reflects its recombination history. The synthetic and the biparental populations presented greater average |D'| and higher frequency of SNPs with elevated |D'| values because they have no recombination history.

Because of the differences regarding molecular marker type and density, sample size, and genome coverage, comparison of LD values of human, domesticated animal, and plant populations should be made with caution, even when the studies involve the same species. We were surprised by the low average r^2^ values and the reduced frequency of SNPs with r^2^ values greater than 0.25 (defined as useful LD in some studies) in the popcorn populations. In the study of Yan *et al*. [30], involving 632 maize inbred lines and 943 SNPs (density of one SNP each 2,121 kb), the average r^2^ was only 0.009. However, for SNPs separated by up to 100 kb, the average was 0.2 (0.03, 0.09, and 0.10 for the biparental, synthetic, and breeding populations, respectively). Even higher LD values were reported in the maize NAM (nested association mapping) population [31] and in two biparental and four FPM (four parent maize) populations studied by Anderson *et al*. [9]. In general, the average r^2^ values observed in the popcorn populations are also lower than the values observed in cattle and chicken populations (0.1 to 0.8 for SNPs separated by up to 100 kb) [32–34]. The density ranged from 27.8 to 112.3 kb in these three studies. Using a 600K SNP chip (density of one SNP per 6.3 kb), Pardo *et al*. [28] observed a median pairwise r^2^ averaged across all chromosomes of 0.015 and 0.016 for the Dutch and HapMap-CEU populations, respectively.

The absence of a uniform criterion for defining the LD decay and the LD extent also makes comparison of the results with human, domesticated animal, and plant populations difficult. Angius *et al*. [26] used LD decay as the distance over which the average LD decreases to half of its maximum value (half-length). They defined LD extent as the distance over which the average LD declines to an asymptotic value. Anderson *et al*. [9] used LD decay as the distance over which the average r^2^ dropped below 0.8, and LD extent as the distance over which the average r^2^ fell below 0.2. Concerning LD decay, our results showed differences between LD measures and populations. There were slight differences between chromosomes, but the higher r^2^ decay occurred after 5–10 kb (36 to 73%). Yan *et al*. [30] observed an LD decay of 64% after 5–10 kb in an inbred lines panel, and the LD reached an approximate asymptotic r^2^ value of 0.01 in the interval of 1–5 Mb (LD extent of 5 Mb). A similar LD extent (5 Mb) was observed in eight breeds of cattle, but a comparable LD decay (62%) occurred along 100 kb [35]. From the analysis of segments of one Mb in all chromosomes in Ashkenazi Jew, caucasian, and African American populations, Shifman *et al*. [36] observed LD decays of 17, 21, and 42% along 10 kb, respectively. A similar LD extent of 300 kb occurred in the populations (reaching an approximate asymptotic r^2^ value of 0.05).

If there is a higher LD between QTLs and haplotypes than with individual SNPs, haplotype blocks can provide substantial statistical power in association studies [6] and increased accuracy of genomic prediction of complex traits [37]. Surprisingly, our results evidenced that the number and length of the haplotype blocks and the number of SNPs per haplotype block were proportional to the average r^2^. The criterion of Gabriel *et al*. [6] appears to provide a reduced number of SNPs per haplotype block. In a study with 235 soybean varieties genotyped by 5,361 SNPs (density of one SNP per 208 kb), Ma *et al*. [38] observed six SNPs per haplotype block on average. This is not surprising because the group of varieties corresponded to a pure line panel (high LD). In studies with German Holstein cattle and four chicken populations, the average number of SNPs per haplotype block ranged between approximately four to 10, and the mean block length ranged from approximately 146 to 799 kb [32, 33]. Low average numbers of SNPs per haplotype block (approximately 4–5) and reduced average haplotype block lengths (approximately 5–7 kb) were also observed in human populations [6, 28]. However, the size of each block varied dramatically in the study of Gabriel *et al*. [6], from less than one to 173 kb.

Concerning the low intragenic LD and the minimum size of the haplotype blocks observed in the three populations, we believe that the lower LD for the biparental population is due to crossing two genetically similar high-quality inbred lines. Because there is no information on the LD and haplotype block patterns in the base populations Viçosa and Beija-Flor, we cannot infer that the higher average intragenic r^2^ values observed in the synthetic and breeding populations (for 11 of the 12 genes) are due to selection for quality. Characterization of the LD and haplotype block patterns regarding specific chromosomal regions has only been made by human geneticists, generally aimed at SNP tagging. From the analysis of SNPs within the HLA region on chromosome 6, Evseeva *et al*. [39] observed 18 haplotype blocks in European populations, based on the criterion of Gabriel *et al*. [6]. Furthermore, the LD was slightly lower in southern than northern European populations. Using the same criterion, Nuchnoi *et al*. [40] observed six and four haplotype blocks across a 472-kb region on chromosome 5q31-33 in Southeast (Thai) and Northeast Asian (Chinese and Japanese) populations. Akesaka *et al*. [41] identified two to six blocks in Korean and Japanese populations, depending on the criterion of an LD block, spanning approximately 3 to 47 kb. The median r^2^ value for the five genes in the region ranged from 0.03 to 0.89.

In conclusion, the level of LD expressed by the r^2^ values in the three popcorn populations with different genetic structures—a biparental population, a synthetic, and a breeding population—is low but comparable to some non-isolated human populations. This finding does not imply that these populations cannot be used for GWAS because there is a fraction of high r^2^ values for SNPs separated by less than 5 kb. The populations are also not excluded for genomic selection because the most important factor affecting this selection process is the relatedness between individuals in the training and validation sets. However, we do not expect a significant advantage from haplotype-based GWAS and genomic selection along generations due to the reduced number of SNPs in the haplotype blocks (2 to 3). The results on LD decay (rapid decay after 5–10 kb) and LD decay extent (along up to 300 kb) are in the range observed with maize inbred line panels. Our most important result is that, similar to human chromosomes, maize (popcorn is also *Zea mays*, but ssp. *everta*) chromosomes also have a pattern of regions with extensive LD (plateaus or cold spots), interspaced with regions of low LD (steps or hot spots). It should be highlighted, however, that our simulated LD map provides evidence that this pattern can reflect regions with differences in allele frequencies and LD level (expressed by D') and not regions with high and low rates of recombination as evidenced by Jeffreys *et al*. [42], since the simulation process assumes a rate of recombination that is proportional to the distance in cM.

## Supporting information

S1 Table Gene name, annotation, and chromosome localization, and the number of intragenic SNPs in each population. (PDF)

S2 Table Minimum and maximum LD values, average distance (kb), and frequency observed in chromosomes by population, concerning SNPs with |D'| and r^2^ values higher than 0.75, in the interval 0.25–0.75, and lower than 0.25. (PDF)

S1 Fig MAF distribution in the biparental population (a), in the synthetic (b), and in the breeding population (c).
(PDF)

S2 Fig LD maps of the populations, by chromosome. (PDF)

S3 Fig LD heatmaps by populations and chromosome regarding the first 100 SNPs; the regions covered ranged from approximately 1.0 to 3.3 Mb; the r^2^ and |D'| values are above and below the diagonal, respectively. (PDF)

S4 Fig LD heatmaps for five segments of 100 SNPs along chromosome 4 in the biparental population; the regions covered ranged from approximately 1.4 to 6.0 Mb; the r^2^ and |D'| values are above and below the diagonal, respectively. (PDF)

S5 Fig Average |D'| values by chromosome and by distance interval (kb) in the biparental population (a), in the synthetic (b), and in the breeding population (c).
(PDF)

S6 Fig Average r2 values by chromosome and by distance interval (kb) in the biparental population (a), in the synthetic (b), and in the breeding population (c).
(PDF)

S7 Fig Distribution of the haplotype blocks based on the number of SNPs in the biparental population (Bip), in the synthetic (Syn), and in the breeding population (BFc4). (PDF)

S8 Fig Overall intragenic |D'| (a, b, c) and r2 (d, e, f) by distance interval (bp) in the biparental population (a and d), in the synthetic (b and e), and in the breeding population (c and f).
(PDF)

S9 Fig Intragenic LD heatmaps by population; the r^2^ and |D'| values are above and below the diagonal, respectively. (PDF)
