# Supplementary material for: Linkage disequilibrium and haplotype block patterns in popcorn populations
Source: PLoS One. 2019 Sep 25;14(9):e0219417. doi: 10.1371/journal.pone.0219417 (PMC6760792; doi:10.1371/journal.pone.0219417)
Supplement: S1 Table — (PDF) [file pone.0219417.s001.pdf]

S1 Table. Gene name, annotation, and chromosome localization, and the number of intragenic SNPs in each population.

Gene	Annotation	Chr.	SNPs	Population
Zm00001d002654	nkd1; naked endosperm1: double mutants have multiple (2-5) layers of peripheral endosperm cells that lack starch granules or other features of starchy endosperm	2	12	Biparental
			7	Synthetic
			7	BFc4
Zm00001d004817	Fatty acid amide hydrolase	2	14	Biparental
			11	Synthetic
			3	BFc4
Zm00001d005451	Cellulose synthase A catalytic subunit 5 [UDP-forming]	2	5	Biparental
			8	Synthetic
			6	BFc4
Zm00001d041972	Cellulose synthase-like protein G3	3	15	Biparental
			9	Synthetic
			10	BFc4
Zm00001d052263	Starch synthase 2 chloroplastic/amyloplastic	4	6	Biparental
			10	Synthetic
			6	BFc4
Zm00001d018033	Starch synthase IIb-2	5	19	Biparental
			6	Synthetic
			5	BFc4
Zm00001d035760	zp15; zein protein, 15kDa15: high methionine; genomic blot indicates one or two copies	6	5	Biparental
			2	Synthetic
			2	BFc4
Zm00001d036900	Cellulose synthase A catalytic subunit 7 [UDP-forming]	6	19	Biparental
			15	Synthetic
			8	BFc4
Zm00001d021731	Cellulose synthase-like protein D3	7	7	Biparental
			9	Synthetic
			9	BFc4
Zm00001d023810	Putative cellulose synthase-like family protein	10	5	Biparental
			12	Synthetic
			16	BFc4
Zm00001d025201	fab1; fatty acid biosynthesis1: endosperm cDNA 2C01H08 (uaz99) similar to fatty acid biosynthesis enzyme	10	9	Biparental
			5	Synthetic
			21	BFc4
Zm00001d026113	nkd2; naked endosperm2: double mutants have multiple (2-5) layers of peripheral endosperm cells that lack starch granules or other features of starchy endosperm	10	20	Biparental
			8	Synthetic
			11	BFc4
