# Supplementary material for: Linkage disequilibrium and haplotype block patterns in popcorn populations
Source: PLoS One. 2019 Sep 25;14(9):e0219417. doi: 10.1371/journal.pone.0219417 (PMC6760792; doi:10.1371/journal.pone.0219417)
Supplement: S2 Table — (PDF) [file pone.0219417.s002.pdf]

S2 Table. Minimum and maximum LD values, average distance (kb), and frequency observed in chromosomes by population, concerning SNPs with |D'| and r^2 values higher than 0.75, in the interval 0.25-0.75, and lower than 0.25.

Pop.	LD value	|D'|		Av. dist.		Freq.		r^2		Av. dist.		Freq.	
		Min.	Max.	Min.	Max.	Min.	Max.	Min.	Max.	Min.	Max.	Min.	Max.
Bip.	>0.75	0.99	1.00	221.5	227.3	0.65	0.74	0.87	0.91	79.7	126.3	0.002	0.005
	0.25-0.75	0.45	0.47	229.4	237.1	0.10	0.17	0.46	0.50	209.5	225.4	0.017	0.048
	<0.25	0.14	0.16	228.0	234.2	0.16	0.18	0.00	0.01	224.9	230.2	0.947	0.981
Syn.	>0.75	0.98	0.99	208.5	222.7	0.61	0.65	0.95	0.96	6.5	19.4	0.008	0.015
	0.25-0.75	0.48	0.49	224.4	238.5	0.22	0.25	0.40	0.41	126.0	153.6	0.024	0.043
	<0.25	0.12	0.13	238.5	249.9	0.13	0.15	0.02	0.02	222.3	235.0	0.943	0.968
BFc4	>0.75	0.98	0.99	206.9	228.9	0.26	0.58	0.93	0.96	5.7	34.8	0.002	0.016
	0.25-0.75	0.44	0.48	219.8	239.3	0.22	0.25	0.41	0.48	19.2	150.1	0.002	0.043
	<0.25	0.10	0.12	230.2	257.4	0.18	0.52	0.01	0.02	223.7	237.1	0.941	0.996
