# Supplementary material for: Linkage disequilibrium and haplotype block patterns in popcorn populations
Source: PLoS One. 2019 Sep 25;14(9):e0219417. doi: 10.1371/journal.pone.0219417 (PMC6760792; doi:10.1371/journal.pone.0219417)
Supplement: S1 Fig — MAF distribution in the biparental population (a), in the synthetic (b), and in the breeding population (c). (PDF) [file pone.0219417.s003.pdf]

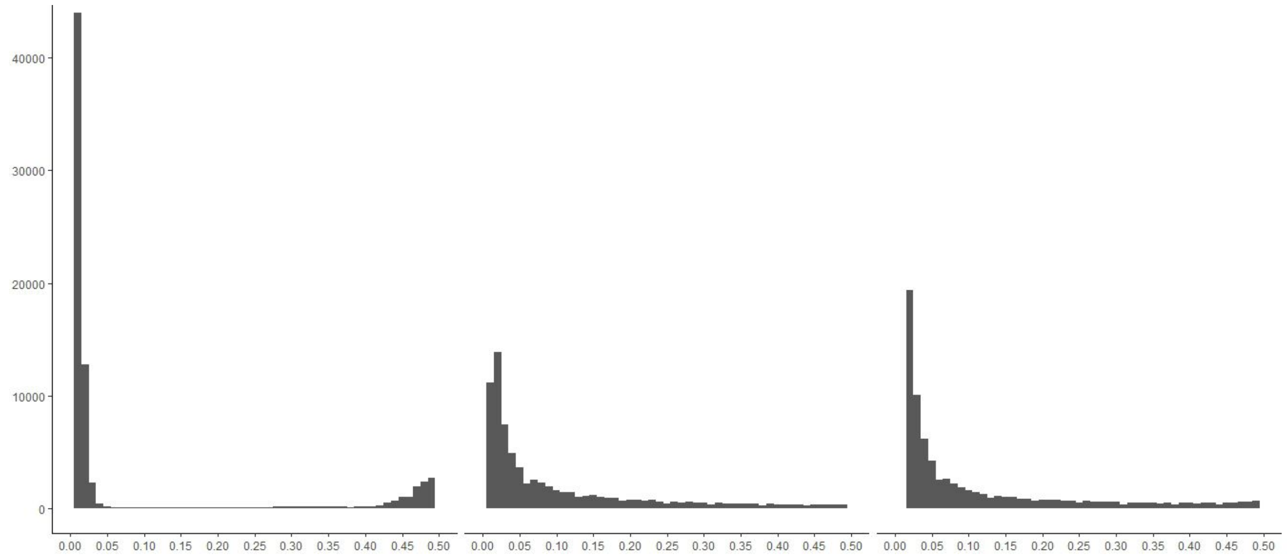

(a) (b) (c)

S1 Fig. MAF distribution in the biparental population (a), in the synthetic (b), and in the breeding population (c).
