# Supplementary figures and images for: Linkage disequilibrium and haplotype block patterns in popcorn populations
Source: PLoS One. 2019 Sep 25;14(9):e0219417. doi: 10.1371/journal.pone.0219417 (PMC6760792; doi:10.1371/journal.pone.0219417)

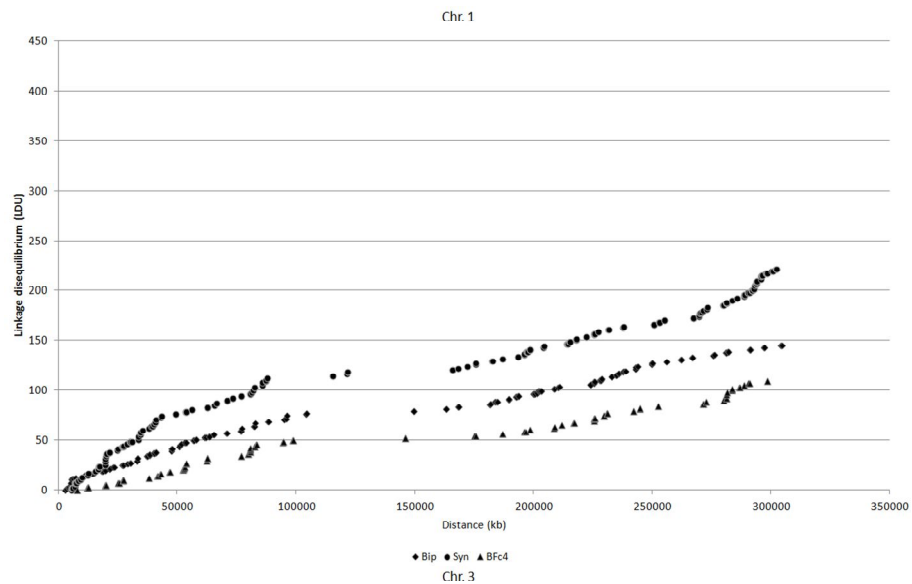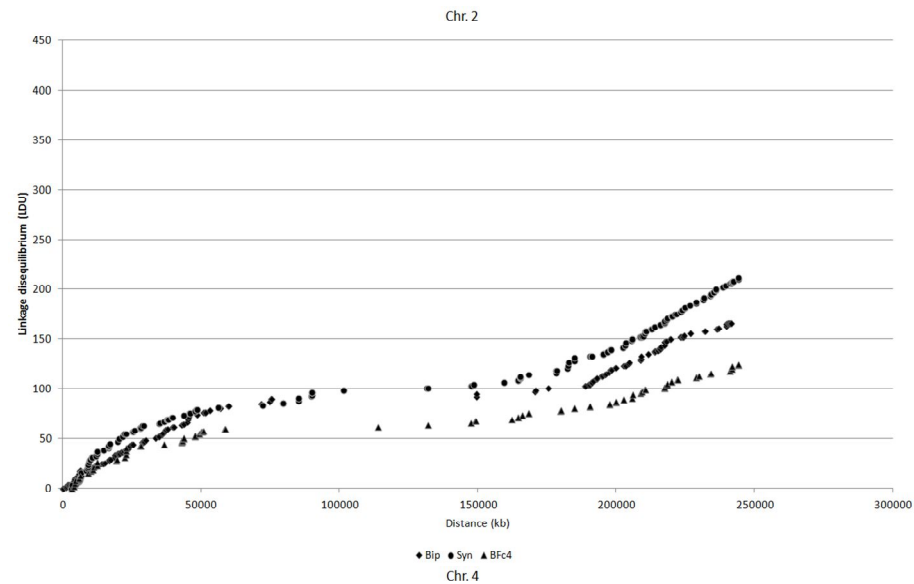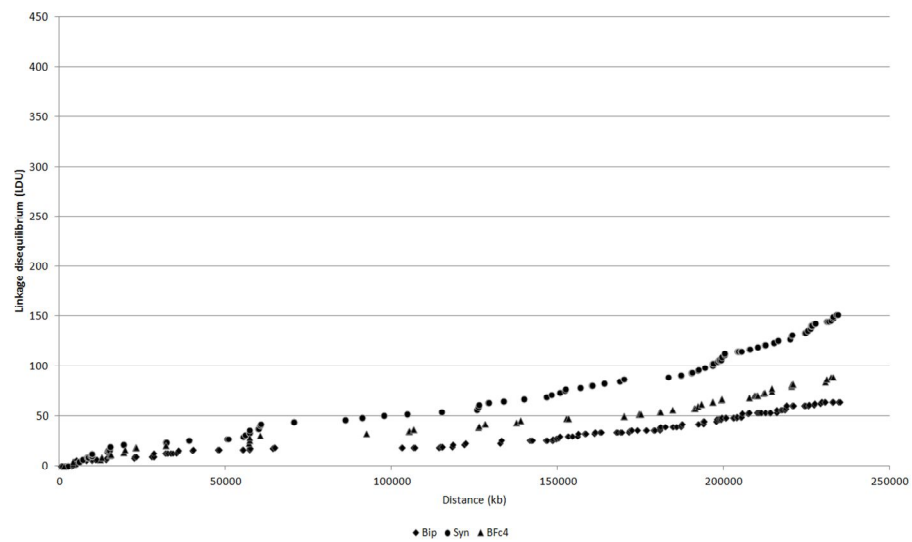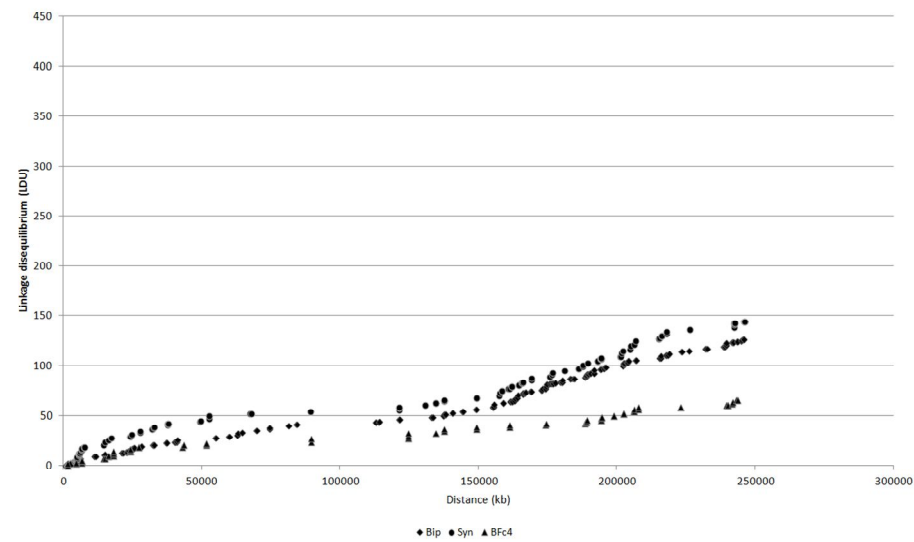

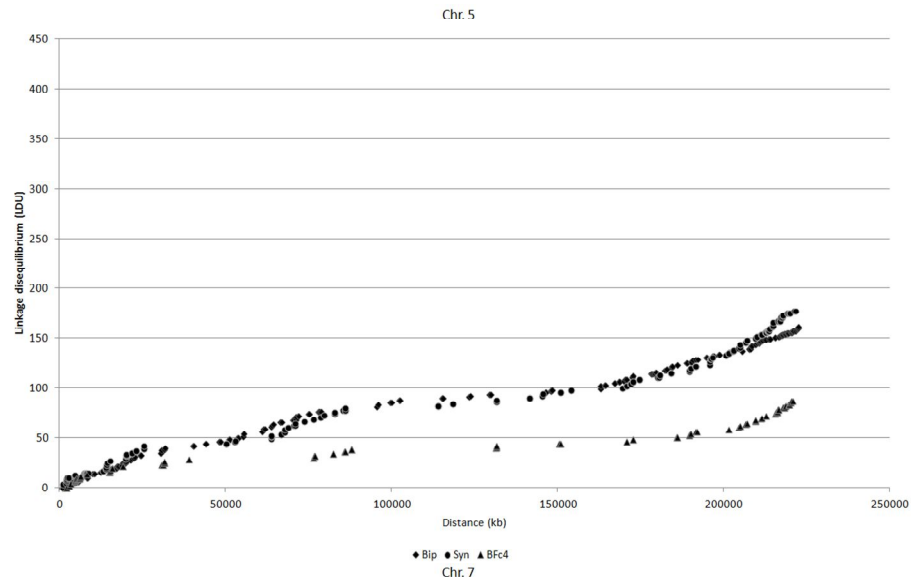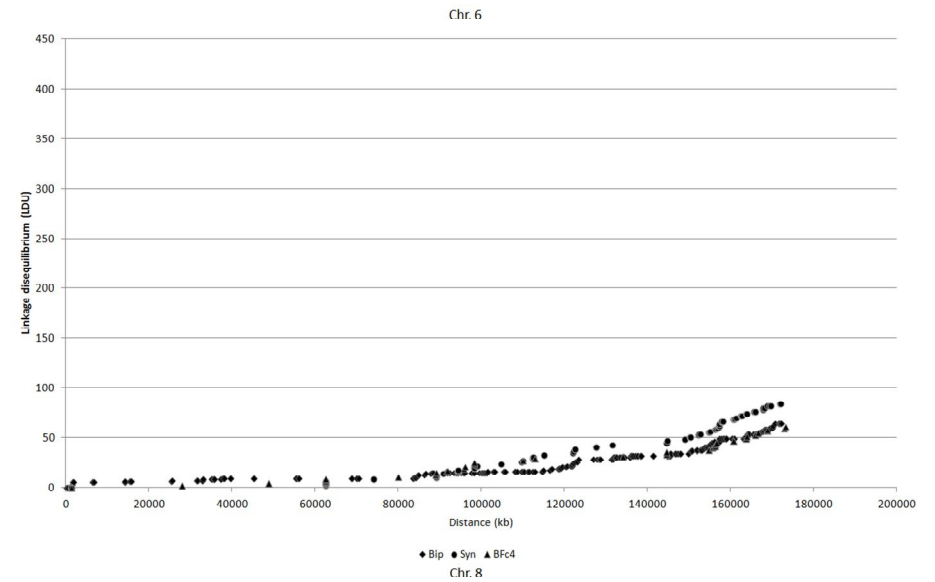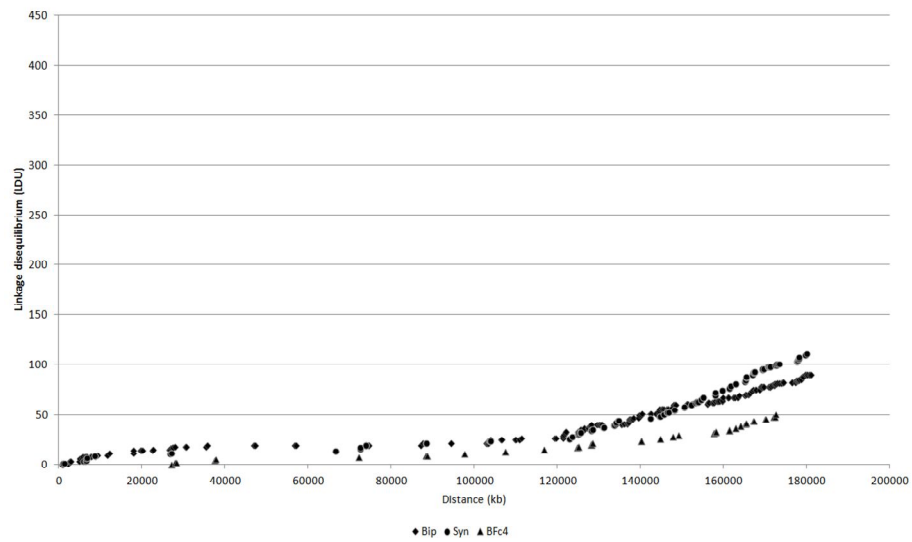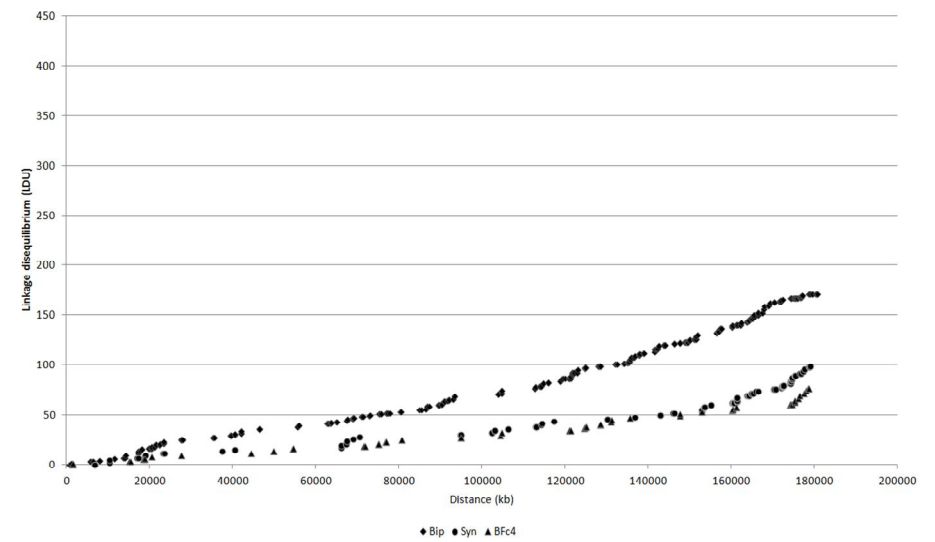

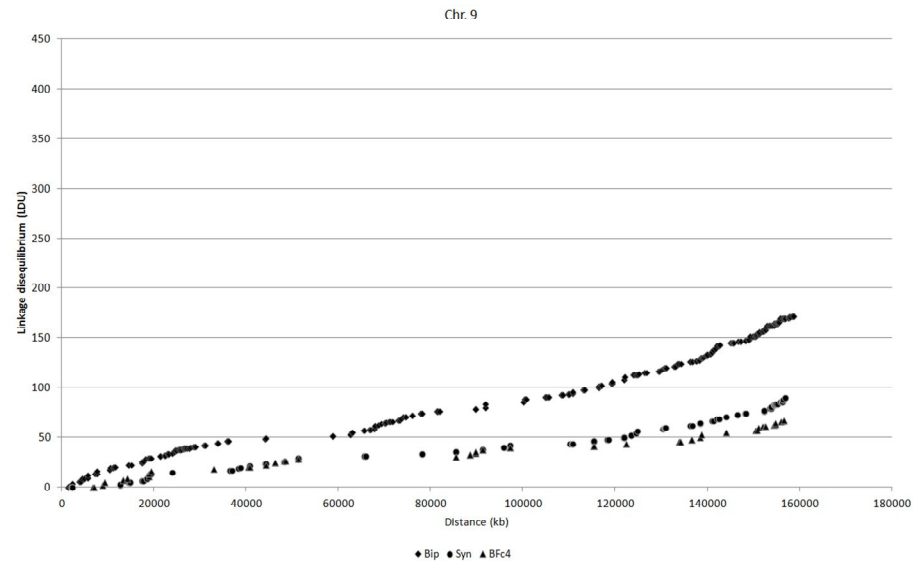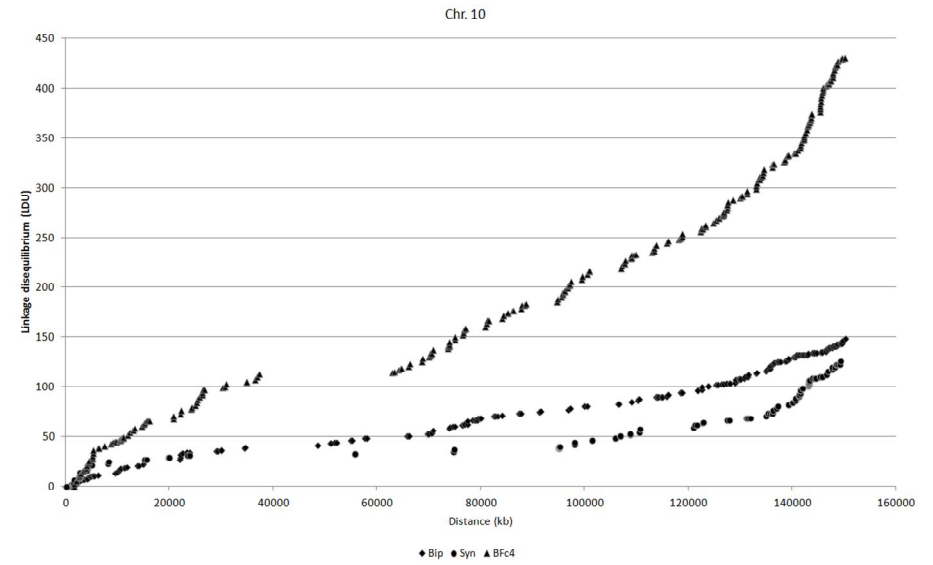

**S2 Fig.** LD maps of the populations, by chromosome.

Supplement: S2 Fig — (PDF) [file pone.0219417.s004.pdf]
