# Supplementary material for: Linkage disequilibrium and haplotype block patterns in popcorn populations
Source: PLoS One. 2019 Sep 25;14(9):e0219417. doi: 10.1371/journal.pone.0219417 (PMC6760792; doi:10.1371/journal.pone.0219417)

S3 Fig. LD heatmaps by population and chromosome regarding the first 100 SNPs; the regions covered ranged from approximately 1.0 to 3.3 Mb; the r^2 and |D'| values are above and below the diagonal, respectively.

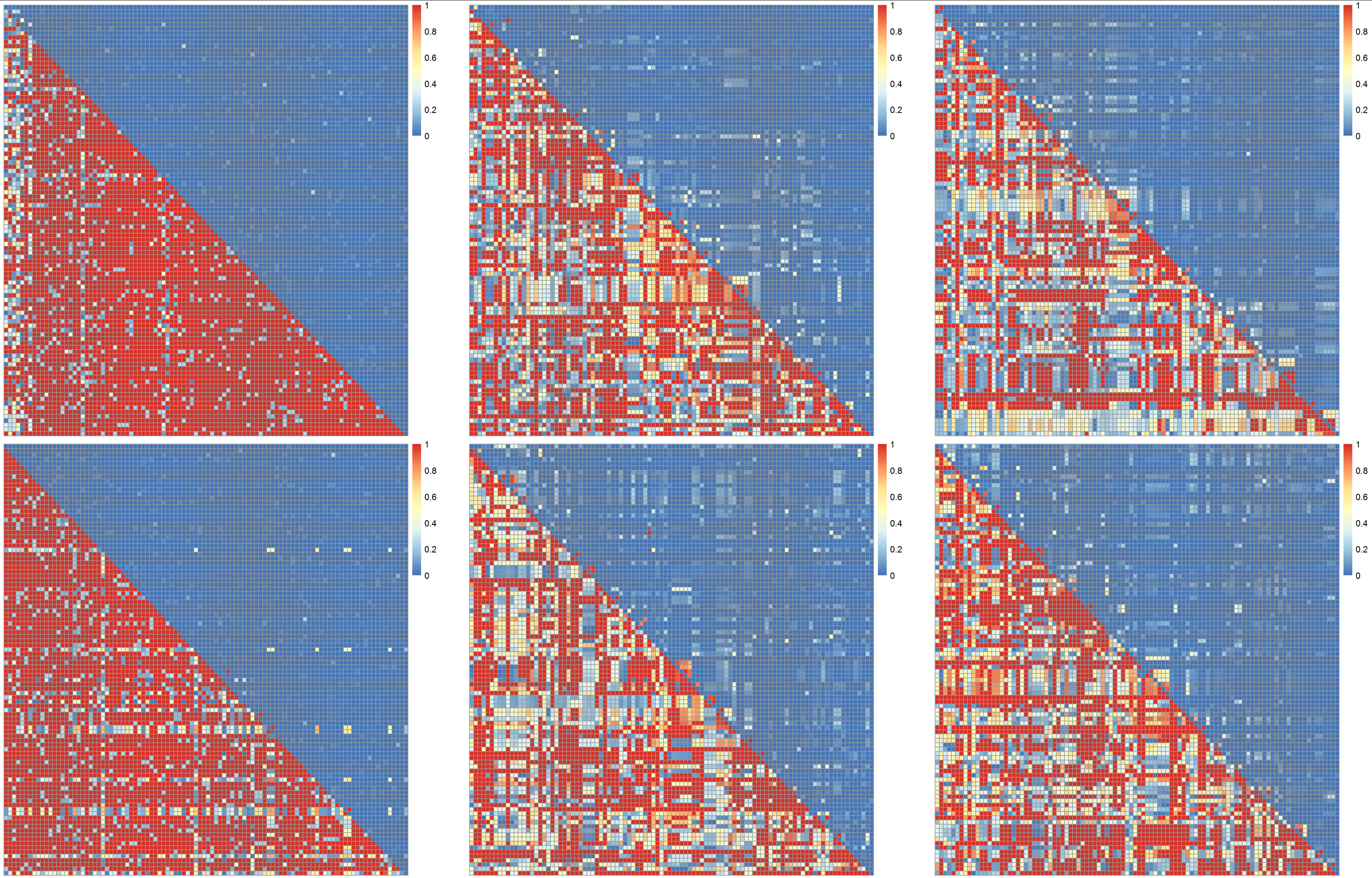

3

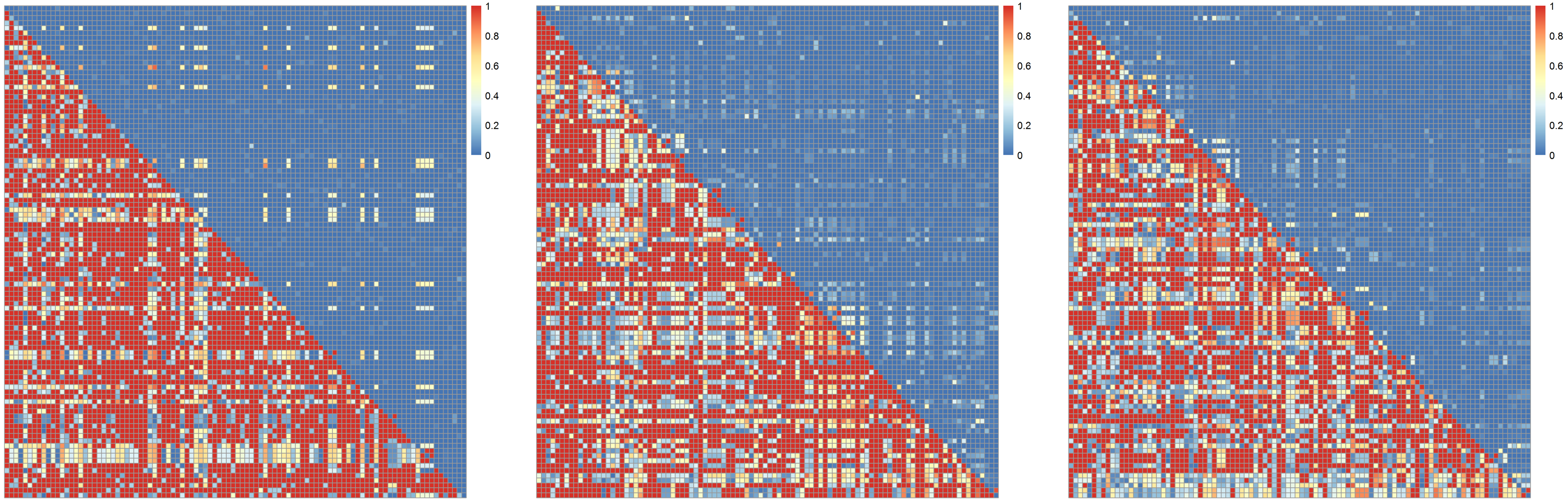

4

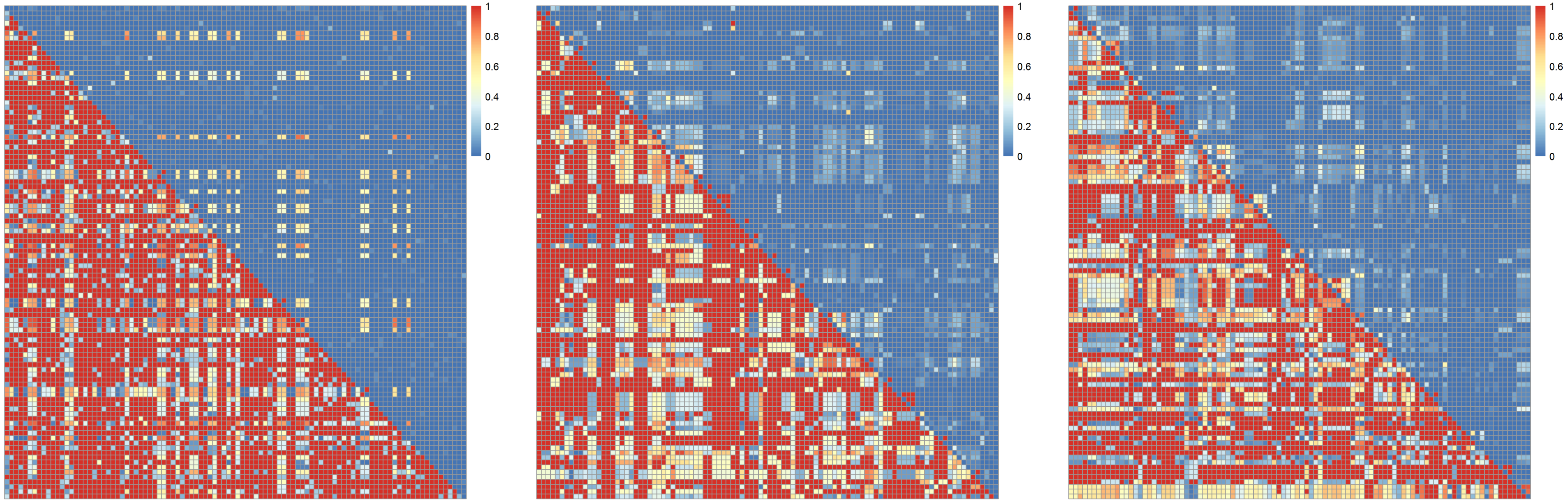

5

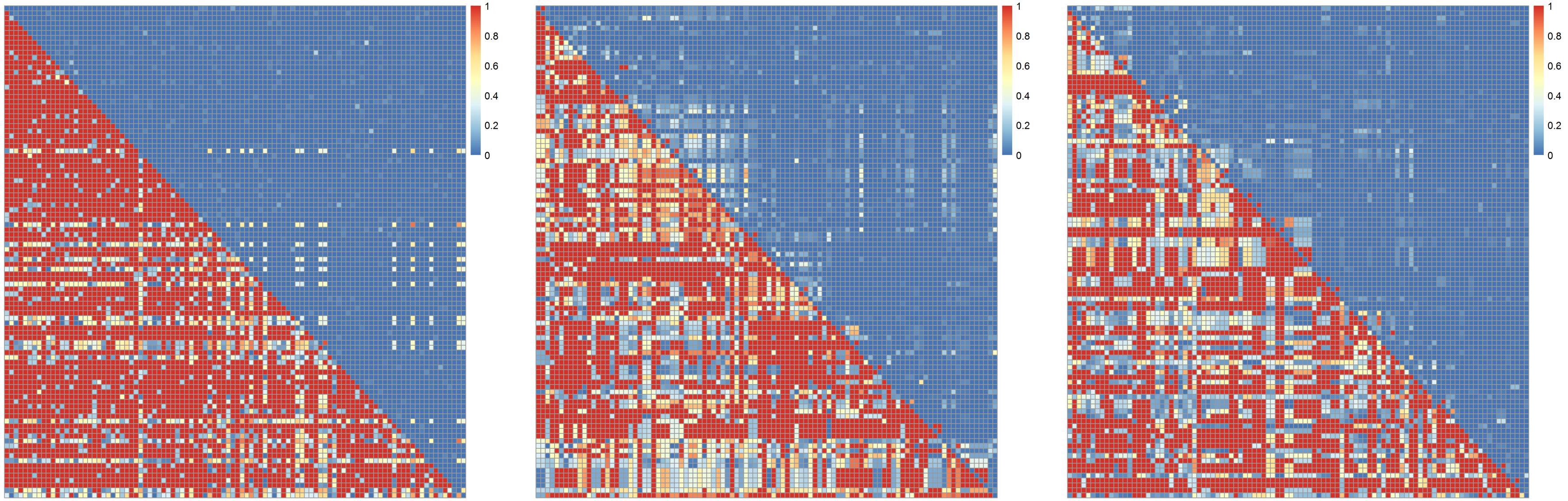
1
0.8
0.6
0.4
0.2
0
1
0.8
0.6
0.4
0.2
0
1
0.8
0.6
0.4
0.2
0

6

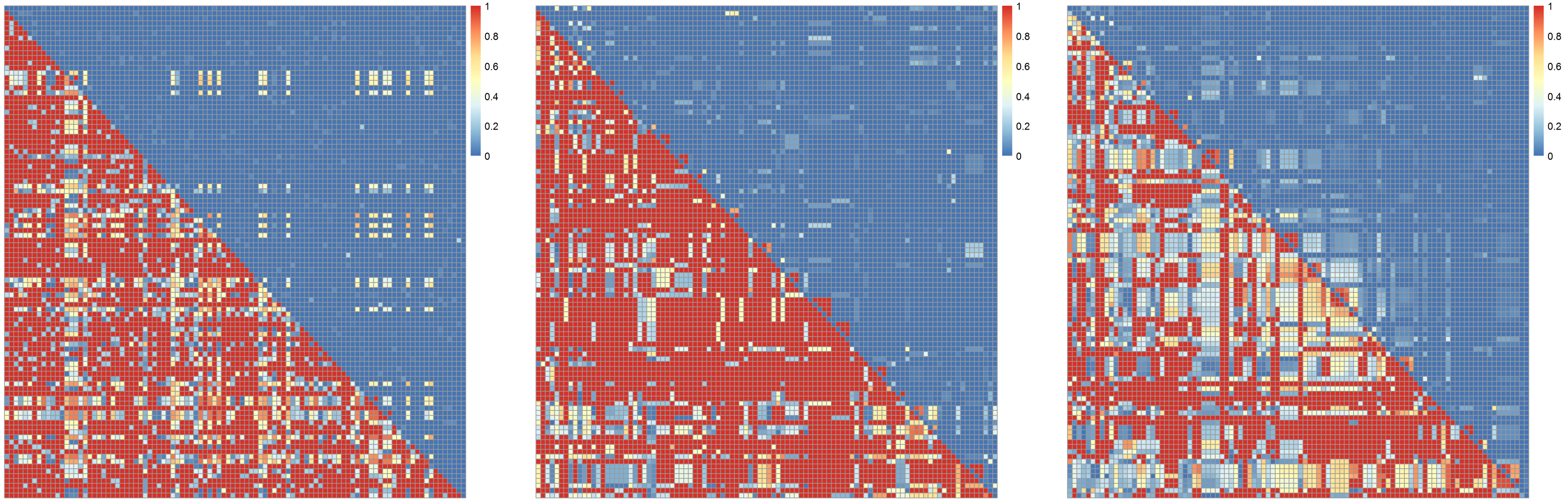
1
0.8
0.6
0.4
0.2
0
1
0.8
0.6
0.4
0.2
0
1
0.8
0.6
0.4
0.2
0

7

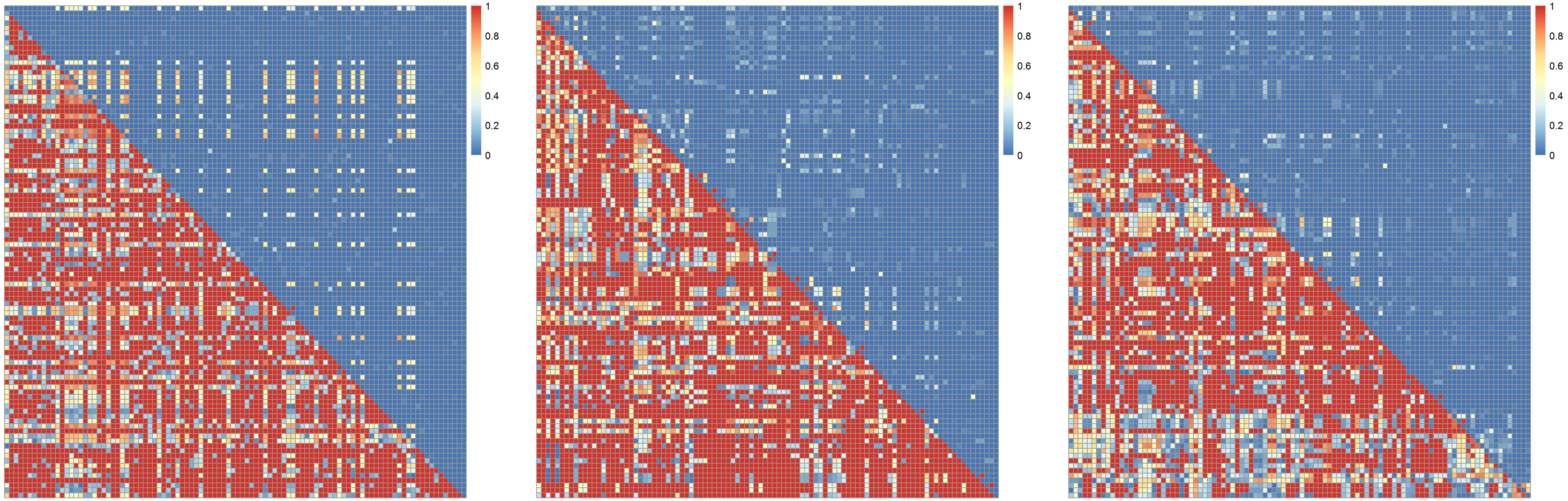

8

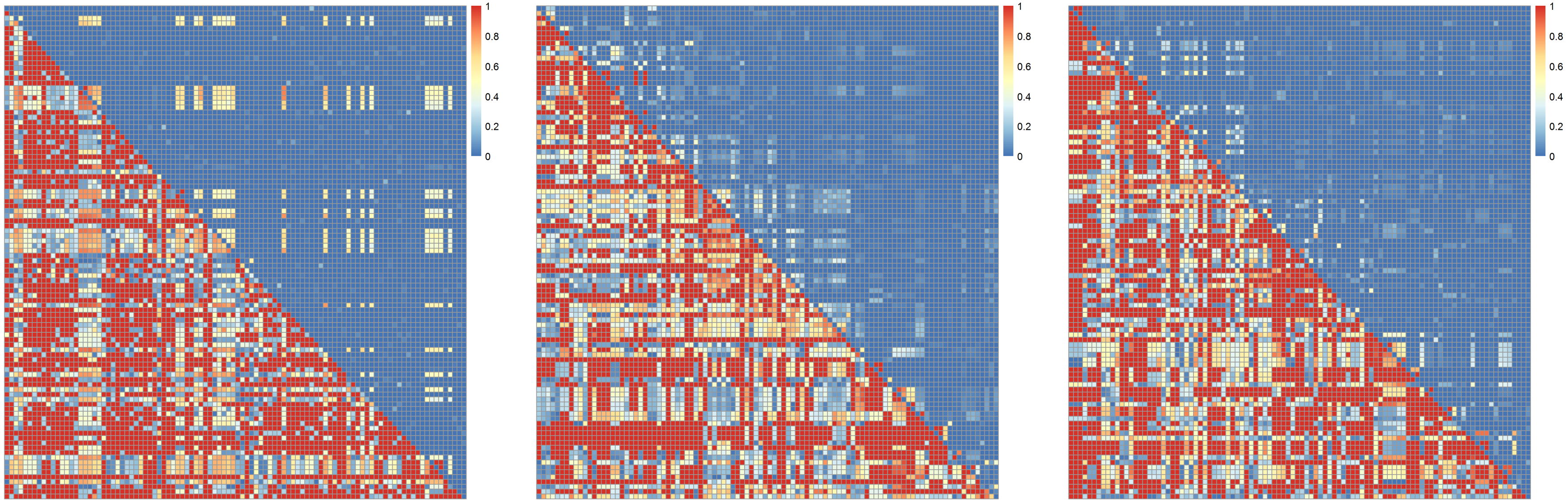

9

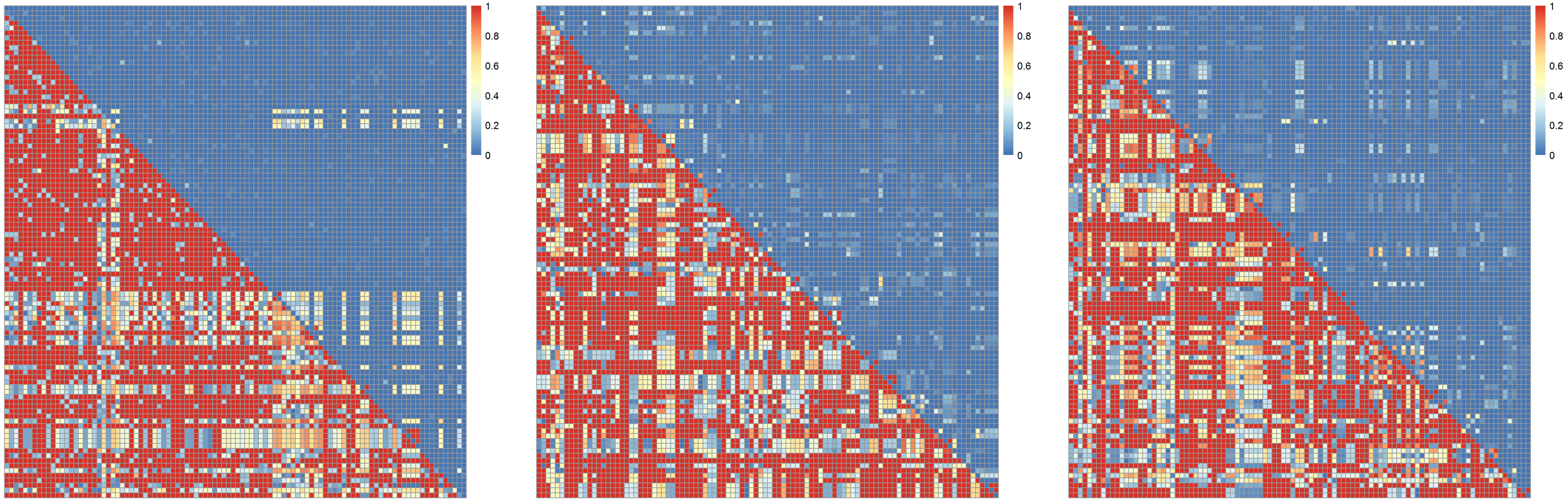

10

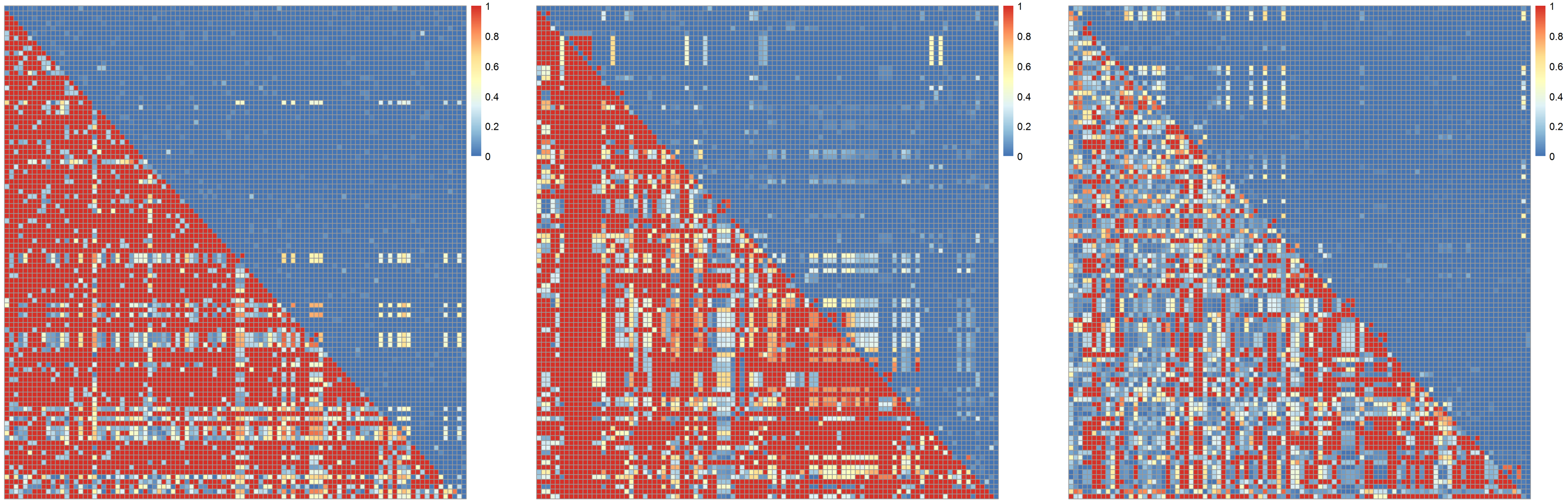

Supplement: S3 Fig — (PDF) [file pone.0219417.s005.pdf]
