# Supplementary material for: Linkage disequilibrium and haplotype block patterns in popcorn populations
Source: PLoS One. 2019 Sep 25;14(9):e0219417. doi: 10.1371/journal.pone.0219417 (PMC6760792; doi:10.1371/journal.pone.0219417)

S4 Fig. LD heatmaps for five segments of 100 SNPs along chromosome 4 in the biparental population; the regions covered ranged from approximately 1.4 to 6.0 Mb; the r^2 and |D'| values are above and below the diagonal, respectively.

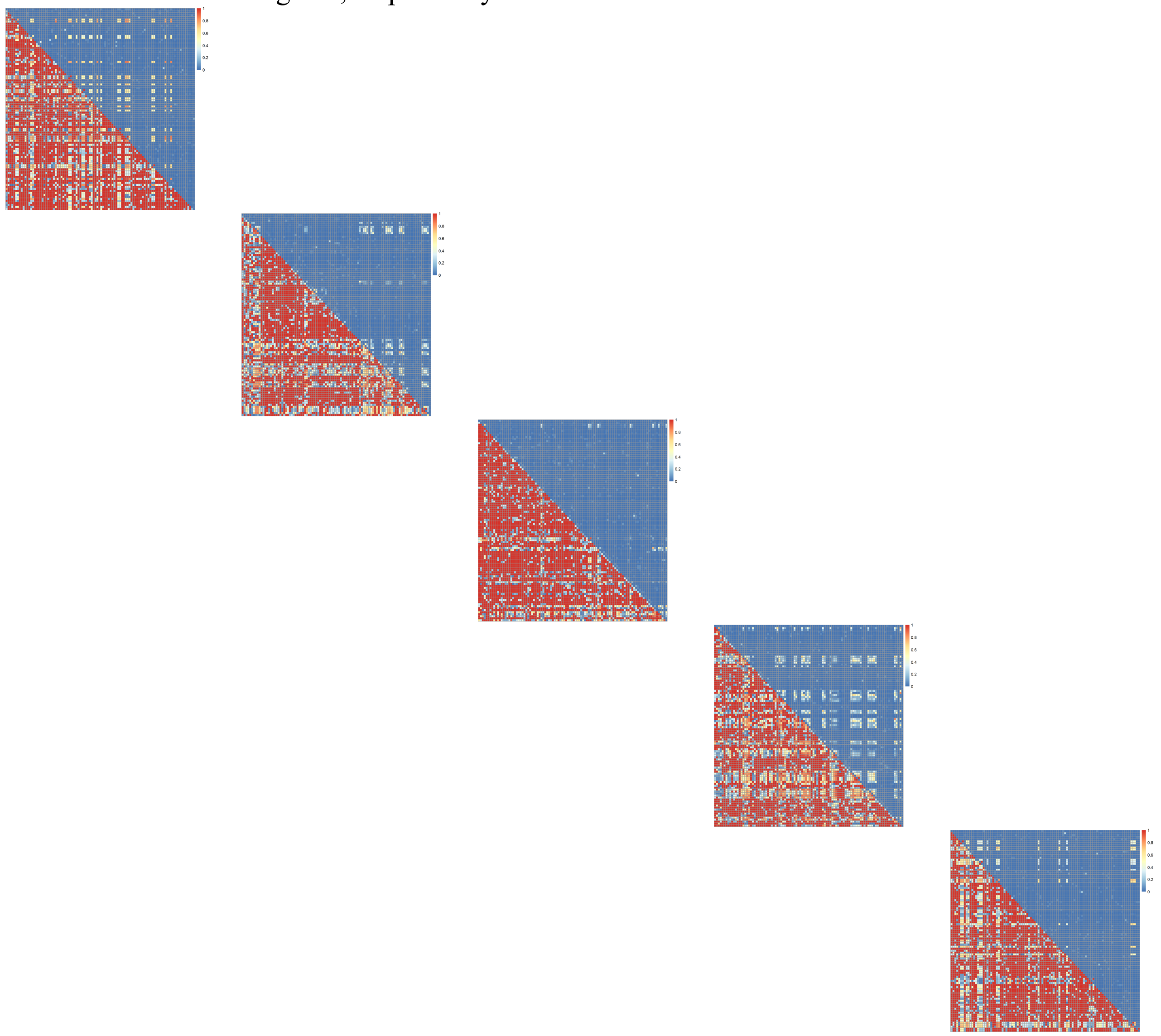

Supplement: S4 Fig — (PDF) [file pone.0219417.s006.pdf]
