# Supplementary material for: Linkage disequilibrium and haplotype block patterns in popcorn populations
Source: PLoS One. 2019 Sep 25;14(9):e0219417. doi: 10.1371/journal.pone.0219417 (PMC6760792; doi:10.1371/journal.pone.0219417)
Supplement: S6 Fig — Average r2 values by chromosome and by distance interval (kb) in the biparental population (a), in the synthetic (b), and in the breeding population (c). (PDF) [file pone.0219417.s008.pdf]

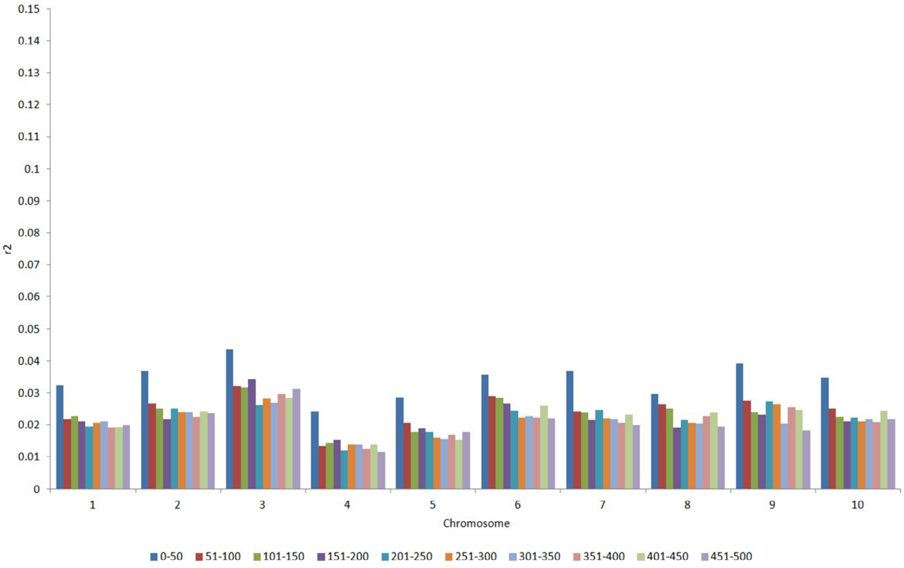

(a)

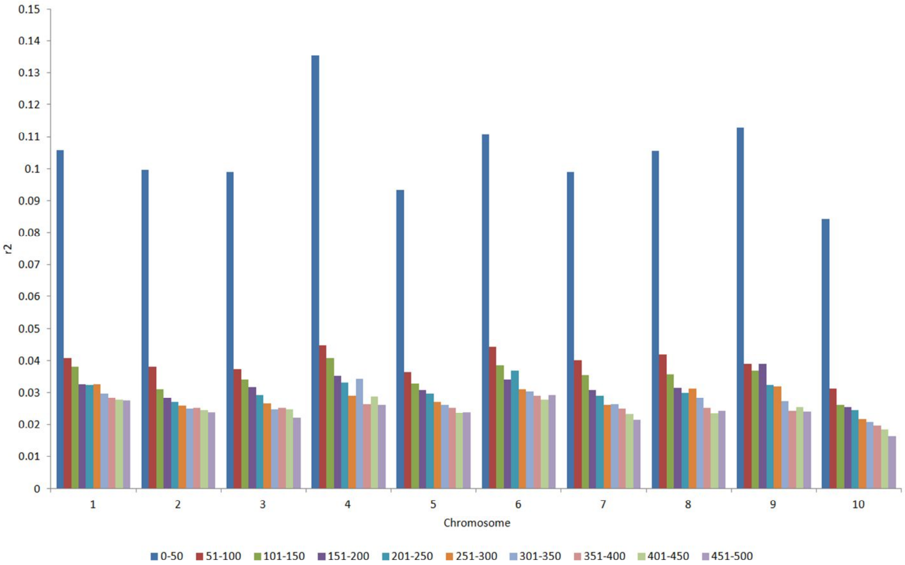

(b)

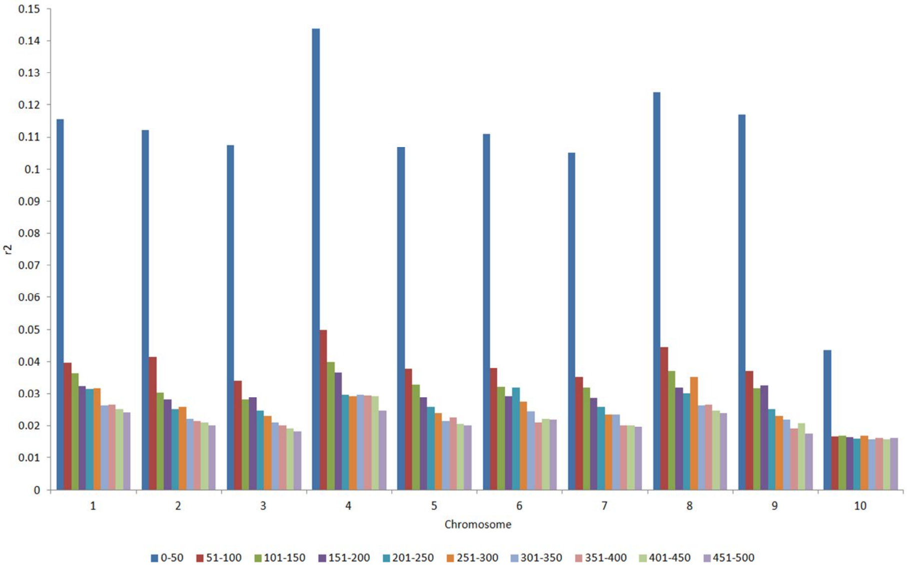

(c)

S6 Fig. Average r^2 values by chromosome and by distance interval (kb) in the biparental population (a), in the synthetic (b), and in the breeding population (c).
