# Supplementary material for: Linkage disequilibrium and haplotype block patterns in popcorn populations
Source: PLoS One. 2019 Sep 25;14(9):e0219417. doi: 10.1371/journal.pone.0219417 (PMC6760792; doi:10.1371/journal.pone.0219417)
Supplement: S7 Fig — (PDF) [file pone.0219417.s009.pdf]

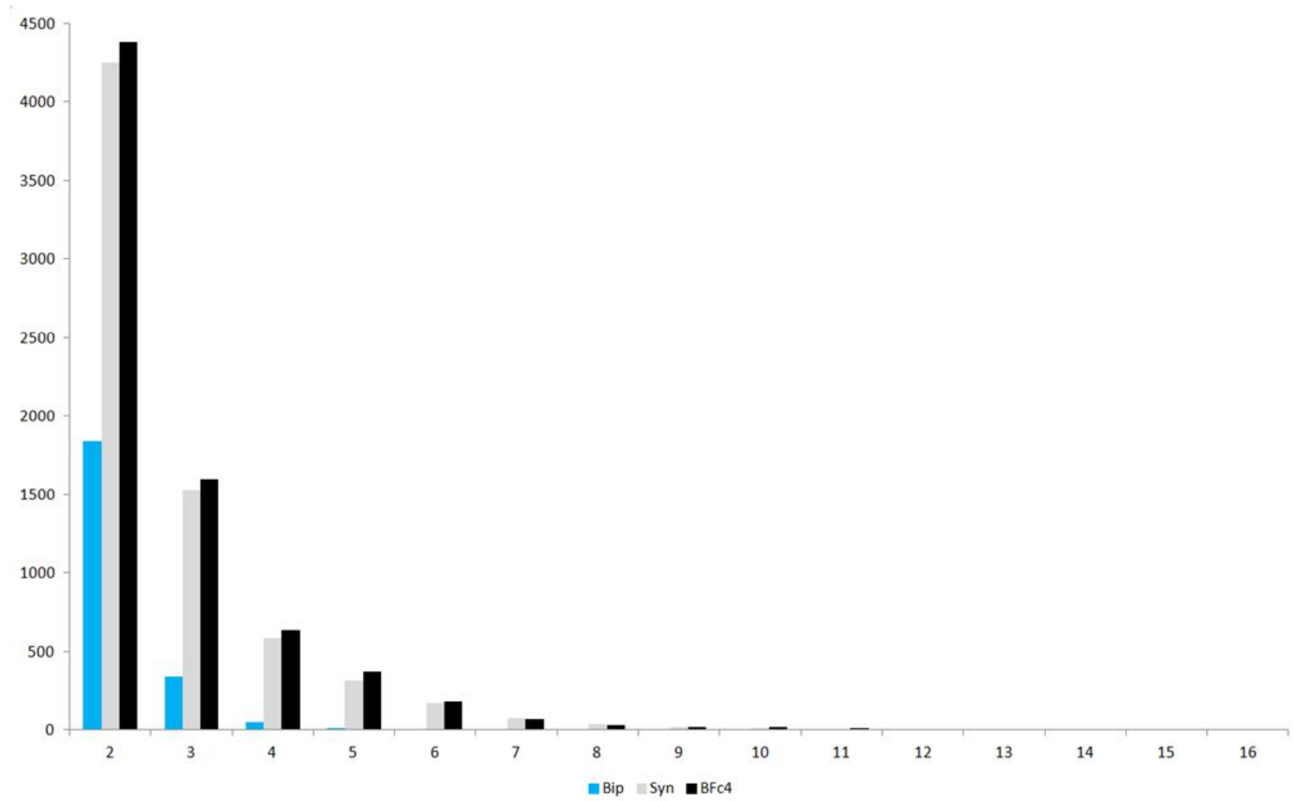

S7 Fig. Distribution of the haplotype blocks based on the number of SNPs in the biparental population (Bip), in the synthetic (Syn), and in the breeding population (BFc4).
