# Supplementary material for: Linkage disequilibrium and haplotype block patterns in popcorn populations
Source: PLoS One. 2019 Sep 25;14(9):e0219417. doi: 10.1371/journal.pone.0219417 (PMC6760792; doi:10.1371/journal.pone.0219417)
Supplement: S8 Fig — Overall intragenic |D'| (a, b, c) and r2 (d, e, f) by distance interval (bp) in the biparental population (a and d), in the synthetic (b and e), and in the breeding population (c and f). (PDF) [file pone.0219417.s010.pdf]

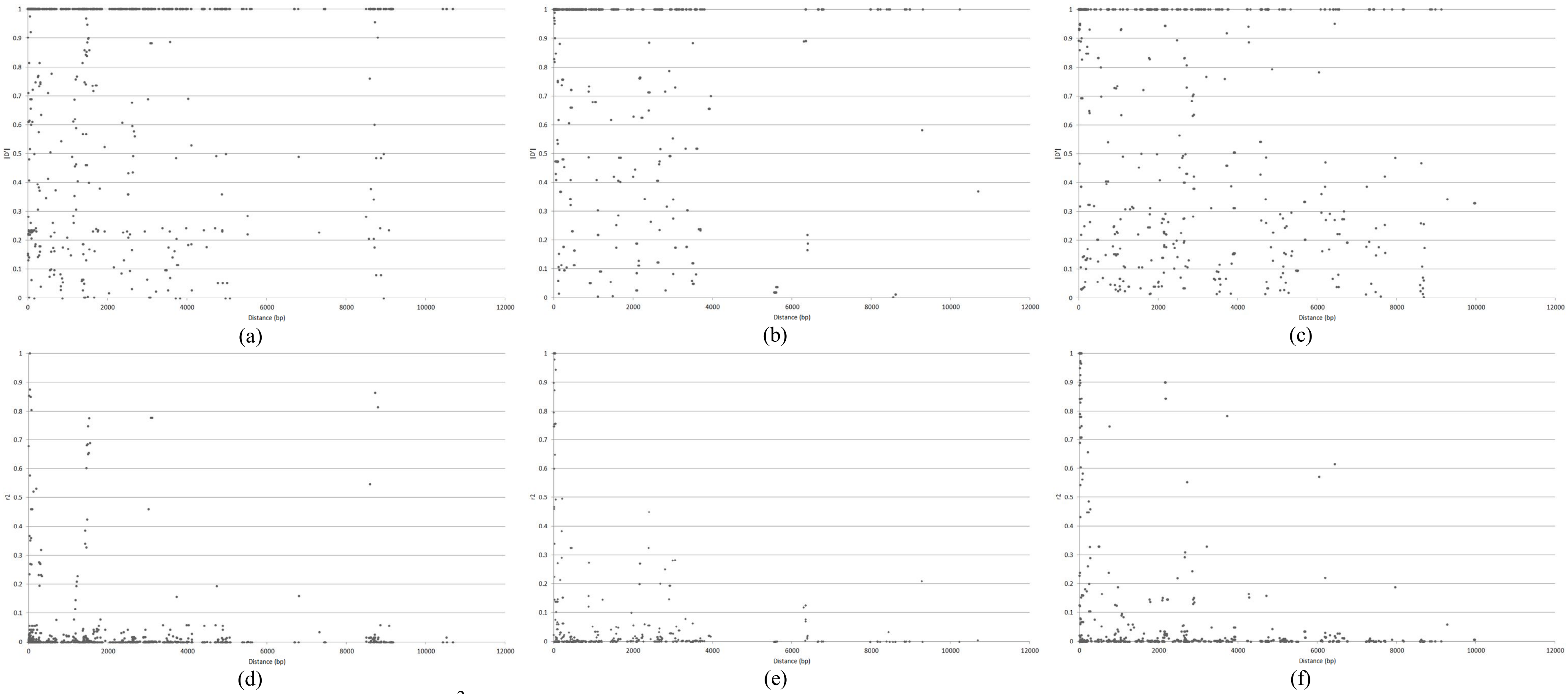

S8 Fig. Overall intragenic |D'| (a, b, c) and r^2 (d, e, f) by distance interval (bp) in the biparental population (a and d), in the synthetic (b and e), and in the breeding population (c and f).
