# Supplementary material for: Linkage disequilibrium and haplotype block patterns in popcorn populations
Source: PLoS One. 2019 Sep 25;14(9):e0219417. doi: 10.1371/journal.pone.0219417 (PMC6760792; doi:10.1371/journal.pone.0219417)

S9 Fig. Intragenic LD heatmaps by population; the r^2 and |D'| values are above and below the diagonal, respectively.

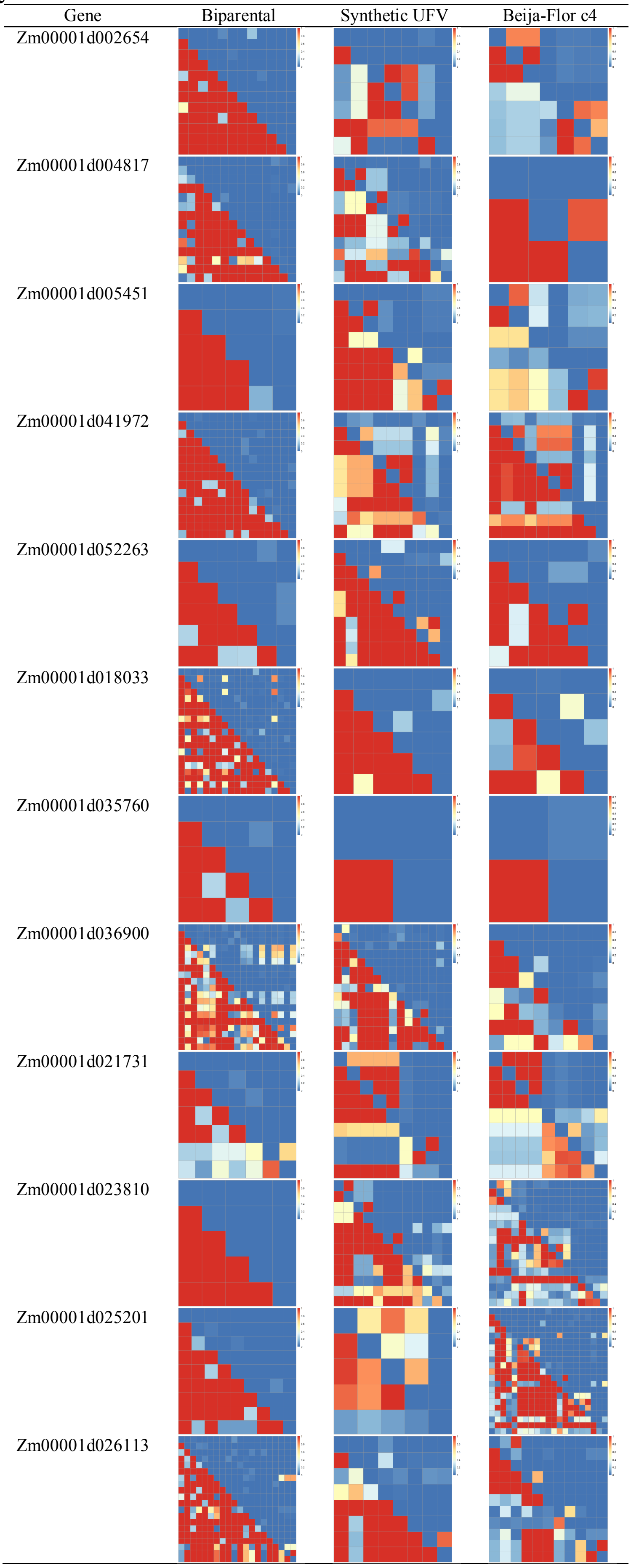

Supplement: S9 Fig — (PDF) [file pone.0219417.s011.pdf]
